# The genomic structure of the highly-conserved *dmrt1* gene in *Solea senegalensis* (Kaup, 1868) shows an unexpected intragenic duplication

**DOI:** 10.1371/journal.pone.0241518

**Published:** 2020-11-02

**Authors:** Ismael Cross, Emilio García, María E. Rodríguez, Alberto Arias-Pérez, Silvia Portela-Bens, Manuel A. Merlo, Laureana Rebordinos

**Affiliations:** Area de Genética, CASEM, Universidad de Cádiz, Puerto Real, Cádiz, Spain; Shanghai Ocean University, CHINA

## Abstract

Knowing the factors responsible for sex determination in a species has significant theoretical and practical implications; the *dmrt1* gene (*Doublesex and Mab-3 (DM)-related Transcription factor 1)* plays this role in diverse animal species. *Solea senegalensis* is a commercially important flat fish in which females grow 30% faster than males. It has 2n = 42 chromosomes and an XX / XY chromosome system for sex determination, without heteromorph chromosomes but with sex proto-chromosome. In the present study, we are providing the genomic structure and nucleotide sequence of *dmrt1* gene obtained from cDNA from male and female adult gonads. A cDNA of 2027 containing an open-reading frame (ORF) of 1206 bp and encoding a 402 aa protein it is described for *dmrt1* gene of *S*. *senegalensis*. Multiple mRNA isoforms indicating a high variable system of alternative splicing in the expression of *dmrt1* of the sole in gonads were studied. None isoforms could be related to sex of individuals. The genomic structure of the *dmrt1* of *S*. *senegalensis* showed a gene of 31400 bp composed of 7 exons and 6 introns. It contains an unexpected duplication of more than 10399 bp, involving part of the exon I, exons II and III and a SINE element found in the sequence that it is proposed as responsible for the duplication. A mature miRNA of 21 bp in length was localized at 336 bp from exon V. Protein-protein interacting networks of the *dmrt1* gene showed matches with *dmrt1* protein from *Cynoglossus semilaevis* and a protein interaction network with 11 nodes (*dmrt1* plus 10 other proteins). The phylogenetic relationship of the *dmrt1* gene in *S*. *senegalensis* is consistent with the evolutionary position of its species. The molecular characterization of this gene will enhance its functional analysis and the understanding of sex differentiation in *Solea senegalensis* and other flatfish.

## Introduction

Knowing the factors responsible for sex determination in a species has significant theoretical and practical implications. First, it enables populations and invasive species to be monitored and managed better; and second, from an applied point of view, it is essential for implementing genetic improvement programs and obtaining monosex populations; this is especially useful when one sex grows significantly faster and/or larger than the other, as is the case with many fish [1, 2].

Chromosomal sex-determination systems appeared in vertebrates 180 million years ago and facilitated rapid adaptive radiation in species; however, the mechanisms of sex control vary widely among species [3]. Whereas *Sry* (Sex determinant Y region) is considered the key regulator of sex determination in most mammals [4], in non-mammal vertebrates a common master regulator of sex has not been found, although there are conserved genes such as *Sox9* (*SRY-box 9*) and *FoxL2 (Forkhead box L2)* that act in a similar way in the regulation of sexual differentiation in all of them [5].

Sex determination systems are especially variable in fish, and can be influenced by environmental factors (ESD) such as temperature, social environment, presence of contaminants or pH of the water or genetic factors (GSD) [6, 7].

Genetic sex determination (GSD) is produced by the expression of sex-determining master genes at the top of gene cascades whose hierarchical expression will lead to the appearance of male or female gonads. So far six different master genes have been reported as responsible, *dmy* (*DM*-domain gene on the Y chromosome), *gsdf* (Gonadal soma derived factor), *sox3* (SRY-box transcription factor 3), *amhy* (Y chromosome-linked anti-Müllerian hormone), *amhr2* (anti-Müllerian hormone type II receptor) and *sdY* (sexually dimorphic on the Y-chromosome) [6]. The heterotermy and hence the dependence of body temperature on environment temperature in fishes, affecting gene expression in the cascade and multistage pathway of biochemical reactions, the lack of natural barriers in the aquatic environment, as well as external fertilization and events of polyploidization during evolution in most fish, have been proposed as the main causes responsible for this variability in systems of gene differentiation in fishes [1].

The first sex-determining master gene described in fish is the *dmy*; this was reported in two species of *Oryzias*, *O*. *latipes* and *O*. *curvinotus*, and originated from a duplication of the autosomal gene *dmrt1*. The duplication occurred 10 to 18 million years ago within the interval of separation of the species *Oryzias mekongensis*, *O*. *latipes*, *O*. *curvinotus a*nd *O*. *luzonensis* [8].

The gene *dmrt1* (*Doublesex and Mab-3 (DM)-related Transcription factor 1*) belongs to a family characterized for possessing a highly-conservative zinc-finger DNA-binding motif (DM domain) rich in cysteines; this zinc-finger contacts predominantly at the minor groove level of the double helix of DNA. This gene has been found to play a vital role in sex determination, differentiation and maintenance of organ functions in a variety of species, including fish, mammals, reptiles, birds and amphibians [9].

The gene d*mrt1* was discovered in mammals; it was the first described case of sex-regulating genes in both vertebrates and invertebrates [10]. In placental and marsupial mammals, *dmrt1* is autosomal and is located "downstream" from the male master determinant gene *Sry*, whereas in mammals of the genus *Platypus* (Order Monotremas), the *dmrt1* gene is located on a sex chromosome and likely [11].

In birds, *dmrt1* is a male sex-determination gene that resides on the Z sex chromosome. The higher dose of *dmrt1* drives testicular development in ZZ birds, while ZW birds lack sufficient dmrt1 to induce male fate, and develop ovaries [3].

In reptiles, which have temperature-dependent sex determination mechanisms, *dmrt1* expression has been detected in the early stages of gonadal development in both sexes, although expression is significantly higher in embryos incubated at temperatures that determine male sex [12]. *Dmrt1* is also involved in testicular development in amphibians. The gene has been found in interstitial cells and Sertoli cells in the testicles one month after metamorphosis, as well as in germ cells of adult individuals, but has not been detected at any stage of ovarian development [13].

Fish display a wide range of different types of sex determination from gynogenesis and different variants of hermaphroditism, together with more frequent gonochoric reproduction, and the determination of sex from the environment, to the genetic: almost all forms of genetic determination of sex have been described in teleost fishes [6].

In most fish species, *dmrt1* mRNA is specifically transcribed in the gonads, and no *dmrt1* transcript was detected in the somatic tissues examined [14–16]. The expression of the *dmrt1* gene is observed in both gonads in species such as the rainbow trout *Oncorhynchus mykiss* [17], zebrafish *Danio rerio* [18], eel *Monopterus albus* [19], the white-tailed eel *Monopterus albus* [18], the silverside *Odontesthes bonariensis* [20], and the Chinese sturgeon *Acipenser cinensis* [21]. However, there is great variability in its expression: a pattern of expression restricted to testicular tissue has been described in species such as medaka *Oryzias latipes* [22], catfish *Clarias gariepinus* [23] and Gibel carp *Carassius gibelio* [24]; whereas in Pegnze carp *C*. *auratus* [25] it is expressed only in the ovaries.

*Solea senegalensis* (Kaup, 1858) is a flat fish that belongs to the order of the Pleuronectiformes. It is a species that naturally presents high growth rates; notably, females grow 30% faster than males [26]. Under aquaculture, however, the species faces several problems, mainly related to reproduction, and the reproduction of individuals in captivity depends exclusively on reproducers of wild origin. Because of the differential growth rates of the sexes and the need for a synchronous and reliable maturation in aquaculture systems, it is essential to understand and control the reproduction processes that are fundamental for efficient propagation.

*S*. *senegalensis* has 42 chromosomes and an XX / XY chromosome system for sex determination, although no heteromorph chromosomes nor sex master-gene has been found [27, 28]. Several cytogenetic studies suggest that the largest metacentric pair of chromosomes could be a proto-sex chromosome pair [29], as they contain the gene cluster *dmrt1-dmrt3-dmrt2*. In addition, evidence based on evolution of the histone genes families has clarified the origin of these two chromosomes as a result of the fusion of 2 acrocentric ones. This is a normal strategy for the evolution of sexual chromosomes [30] and would explain the differences with respect to the chromosomes observed in the two closely-related species *Scophthalmus maximu*s and *S*. *rhombus*, which present a karyotype with 2n = 44 chromosomes including two metacentric pairs [31]. The origin of this chromosome has also been confirmed by Zoofish data obtained from two species of the Soleidae family (*Dicologlossa cuneata* and *Dagetichthys lusitanica*), and by comparative genome analysis with *Cynoglossus semilaevis* [32].

It is known that repetitive DNA accumulation has the potential to initiate the evolution of sex chromosomes [33]; and the analysis of repetitive elements throughout the entire genome of *S*. *senegalensis* shows a greater presence of this sequences in chromosome 1, as well as specific families related to sex chromosomes [34]. In particular, the number of retroelements and simple repeats per region shows its highest value in the subcentromeric region of the chromosome on which the *dmrt* gene family is localized. This region contains all the *dmrt* genes, which are associated with sex determination in some species [35]. Hence a detailed analysis of the *dmrt1* gene could contribute to the discovery of the master sex gene in the species.

In the present study, we report the genomic structure of *dmrt1* gene, based in the analysis of transcripts isolated from the gonads of the sole. The molecular characterization of this gene will enhance its functional analysis and hence the sex development of *S*. *senegalensis* and flatfish.

## Materials and methods

### Animal use and tissue sample preparation

Samples of *S*. *senegalensis* aged five years were obtained from a population bred artificially in the hatchery of the Central Marine Cultivation Research Service (SC-ICM) in the Andalusian Centre of Marine Studies (CASEM, University of Cadiz). A pair of adult specimens were obtained in a naturally-occurring population in the southwest of Iberian Peninsula (Bay of Cadiz). A total of nine adults of *S*. *senegalensis* (5 males and 4 females) were used; the male average lengths were 40,3 +/- 0,06 cm and the female average lengths 43,2 +/- 0,06 cm. The average weight were 1028 +/- 141 g in males and 1408 +/- 189 g in females. Fish were transported in oxygenated containers to the laboratory for analysis. The fish were anesthetized with 90–100 mg/l of clove oil and dissected. The gonad samples were carefully removed and stabilized by immersing them immediately in RNAlater® TissueProtect (Qiagen), and then stored at -80°C before RNA extraction.

The experimental procedures were in accordance with the recommendation of the University of Cádiz (Spain) for the use of laboratory animals (https://bit.ly/2tPVbhY) and the Guidelines of the European Union Council (86/609/EU). The experiment was authorised by the Ethics Committee of University of Cadiz (Spain).

### RNA isolation and cDNA synthesis

RNA extraction was carried out with the RNeasy Tissue Mini Kit and the lysis reagent QIAzol Lysis Reagent (Qiagen) following the manufacturer's instructions. RNA quality and concentration were evaluated by measuring the A260 / A280 ratio using a NanoDrop 2000c spectrophotometer (ThermoScientific). The synthesis of complementary DNA (cDNA) was carried out using the iScript gDNA Clear cDNA Synthesis Kit (Bio-Rad). The cDNA was synthesized from RNA treated with DNase using a reverse transcriptase of the kit: iScript Reverse Transcription Supermix, for 20 min at 46°C. To test *dmrt1* expression in gonads, a pair of primers, *Sse-F1* and *Sse-R1*, were used. All primers used in this work are reported in Table 1.

**Table 1:** Primer sequences used and their role in this study.

Primer	Sequence	Purpose
*Sse-F1*	GCTGCAGGAACCACGGCTACGTGTC	Expression test; BAC screen; exons I- IV cDNA analysis
*Sse-R1*	GGAGGAGGAGCTTGGTATTTGCAGTCC	Expression test; BAC screen
*Sse-5'RACE-R1*	ATCTGAGACCATGGCAGCAT	5’RACE; exons I- IV cDNA analysis
*Sse-3'RACE-F1*	ACCTACTACAGCAACCTCTACAACTACCAGCAATACCA	3’RACE
*Abridged anchor primer (AAP*, *commercial)*	GGCCACGCGTCGACTAGTAC(G)_14_	5’RACE
*Abridged universal amplification primer (AUAP*, *commercial)*	GGCCACGCGTCGACTAGTAC	3’RACE

Expression test: *primers* used to test *dmrt1* expression in RNA extracted from gonads; BAC screen: Primers used to amplify sequences from the BAC containing the *dmrt1* gene.

PCR reactions were carried out using a PCR mixture of 50 μl volume with 2 μl of gonad cDNA, 1 μl of 10 μM of each primer, 10 μl of My Taq Red Buffer, 0.4 μL of My Taq HS (5U/μL) and 35.6 μL of sterile MQ water. The PCR conditions were: 95°C for 3 minutes; 95°C for 15 seconds, 55°C for 15 seconds, and 72°C for 30 seconds, for 35 cycles, plus an additional prolongation at 72°C for 10 minutes in the last cycle. PCR fragments were detected by electrophoresis and developed with ethidium bromide.

### 3´ RACE system for rapid amplification of cDNA ends

The 3' RACE (Rapid Amplification of cDNA Ends) reaction was carried out using the 3' RACE system (Invitrogen). The cDNA of the first strand was synthesized from the gonadal RNA using the Superscript II RT reverse transcriptase Kit. For the amplification of the target cDNA by PCR, the *Sse-3’ RACE-F1* primer (Table 1) and the *AUAP* commercial primer were used. A PCR mixture of 50 μl volume was produced with 2 μl of gonad cDNA, 1 μl of 10 μM of each primer (*Sse-3' RACE-F1* and *AUAP*), 10 μl of My *Taq* Red Buffer, 0.4 μL of My Taq HS (5U / μL) and 35.6 μL of sterile MQ water. The PCR was carried out with the PCR contact conditions: 95°C for 3 minutes; 95°C for 15 seconds, 55°C for 15 seconds and 72°C for 30 seconds, for 35 cycles, plus an additional prolongation at 72°C for 10 minutes in the last cycle. PCR fragments of size were detected by electrophoresis and developed with ethidium bromide.

### 5´ RACE system for rapid amplification of cDNA ends

The 5' RACE reaction was performed using the 5' RACE System for Rapid Amplification of cDNA Ends kit (Invitrogen). The first strand of cDNA synthesis was prepared using primers specific for the *dmrt1* gene (Table 2) and the Superscript II RT reverse transcriptase Kit, after adding homopolymer tails to the 3' ends of the cDNA with a terminal deoxynucleotidyl transferase (TdT). A PCR mixture of 50 μl volume was produced with 2 μl of tail cDNA, 1 μl of 10 μM of each primer (*Sse-3' RACE-F1 and AAP*), 10 μl of My *Taq* Red Buffer, 0.4 μL of My *Taq* HS (5U/μL) and 35.6 μL of sterile MQ water. The PCR was carried out with the PCR contact conditions: 95°C for 3 minutes; 95°C for 15 seconds, 55°C for 15 seconds and 72°C for 30 seconds for 35 cycles, plus an additional prolongation at 72°C for 10 minutes in the last cycle. PCR fragments of size were detected by electrophoresis and developed with ethidium bromide. All 5' RACE PCR products were purified using the clean-up Gel extraction PCR kit (Macherey Nagel).

**Table 2:** Variability and nucleotide divergence when comparing intron paralogues, by duplication, in *dmrt1* gene of *Solea senegalensis*.

Region	Length	Number of sites without gaps	Number of sites with gaps	Monomorphic sites	Polymorphic sites	Pi(JC)
Intron 1 and 1’	695	577	118	566	11	0,019
Intron 2 and 2’	2401	2006	395	1674	332	0,187
Intron 3 and its duplicated region in intron 3’	2328	1454	874	1341	113	0,082

Number of sites without and with gaps, monomorphic and polymorphic sites have been calculated. The nucleotide divergence (*Pi* of Jukes and Cantor) between pairs of introns is displayed too.

### Cloning and sequencing of *dmrt1* cDNA

For cloning products obtained by the *dmrt1* cDNA sequencing process, we used the Cloning Protocol with the TOPO ™ TA Cloning Kit for Sequencing, with One Shot ™ TOP10 Chemically Competent *E*. *coli* (Invitrogen) following the manufacturer's instructions. Positive clones were identified by colony PCR using the universal M13 primers and sequenced. The sequences (S1 Sequences) were then analyzed using VecScreen and identified as *dmrt1* transcripts by comparison with existing sequences using the BLAST tool at http://www.ncbi.nlm.nih.gov. The amino acid sequence of the main *dmrt1* protein product was deduced from assembled RACE sequences using an Expasy tool to translate (http://web.expasy.org/translate/). To figure out an alternative splicing process, all cDNA sequences from forty clones were aligned and analyzed using MAFFT (https://mafft.cbrc.jp/alignment/server/) software and BLAST algorithm. The alignments were individually completed and adjusted in the final editing process of the annotation by using the UGENE (https://github.com/ugeneunipro) and BioEdit 7.0.9.0 software https://bioedit.software.informer.com/download/?lang=es.

### *Dmrt1* interaction study

In order to analyze the protein-protein interaction of *dmrt1*, the deduced amino acid sequence was used in a search process in the STRING database (www.string-db.org). This database assigns protein functional enrichment using functional classification systems such as Pfam, InterPro, KEGG and gene ontology (GO), taking information from experimental data and public collections.

To investigate the presence of the putative target for mature miRNA in the cDNA 3’UTR (untranslated region) sequence, the miRNA database (http://www.mirbase.org/) was used. A BLAST search was applied against mature miRNA from teleostei with parameters: Evalue cutoff = 10; Word size = 4; Match score = +5; Mismatch penalty = -4.

### Phylogenetic analysis

In total, 32 amino acid sequences (one of them corresponding to *Xenopus*, included as outgroup) were used. The accession numbers are given in the S1 Table. The SMS (http://www.atgc-montpellier.fr/sms/) program was applied to determine the best-fit phylogenetic model and, finally, the PhyML 3.0 software was used to run the model (https://github.com/stephaneguindon/phyml/releases).

The resulting best-fit model predicted was JTT+G+I+F. The proportion of invariable sites was 0.115; the number of substitution rate categories was 4; and the Gamma shape parameter estimated was 2.158. The statistic used for model selection was the Akaike information criterion (AIC), the value of which was 17290.67 and the -LnL was -8561.34. Branch support was tested by the fast likelihood-based method using aLRT SH-like. Tree edition was carried out using MEGA version 7 (https://www.megasoftware.net/).

### Analysis of the *dmrt1* gene structure in the genome of *S*. *senegalensis*, and its association with repetitive elements

To annotate and describe the exons and introns structure in the *S*. *senegalensis* genome, the sequence of a *S*. *senegalensis* BAC clone containing the *dmrt1* gene (BAC 48K7) was used [32]. The cDNA *dmrt1* sequence was aligned with the BAC sequence using MAFFT software (https://mafft.cbrc.jp/alignment/server/), assisted with BLAST searches. Finally, alignments were manually edited using the bioinformatic tools Bioedit and UGENE. DnaSP v6 (http://www.ub.edu/dnasp/) was used to analyse nucleotide diversity and polymorphism in duplicated introns.

In order to identify *in silico* repetitive elements associated with this gene and to analyze their distribution throughout the BAC sequence, two approaches were applied. First, the assembled sequence of the BAC clone (BAC 48K7) containing the *dmrt1* gene was analyzed using the software Repeat Masker (http://www.repeatmasker.org/cgi-bin/WEBRepeatMasker), to obtain the coordinates in the clone of the different types of repetitive elements: retroelements, DNA transposons, low-complexity sequences and satellites. Second, the raw genomic reads from BAC 48K7 were analyzed using the graph-based clustering approach of the Repeat Explorer (RE) pipeline to characterize repetitive content of BAC 48K7 (www.repeatexplorer.org). RE is a graph-based clustering algorithm that clusters sequences based on their similarity. The algorithm assembles sequences from each cluster and produces contigs that are used as reference sequences, and represent repetitive elements (TEs and satellites) present in the BAC. Before starting the RE pipeline, the BBDuk (from BBTools toolset) program (https://sourceforge.net/projects/bbmap/was applied as a trimming and quality tool to filter the raw reads (454 Life sequencing) from the BAC, with parameters: ktrim = r, k = 23, mink = 11, hdist = 1, qtrim = rl, trimq = 20, ftl = 3, ftr = 700. Repeat Explorer was then used with the following settings: taxon and protein domain database version (REXdb): Metazoa version 3.0; queue: “extra-long and slow”. The results were provided in a HTML archive report and all the data were downloaded in a single archive for further investigation. Then, to check for DNA transposons in the cluster sequences obtained with Repeat Explorer, they were used as input search data in the Dfam database (https://dfam.org/home) with *D*. *rerio* as Query Organism and 0.001 as E-value.

## Results

### *S*. *senegalensis dmrt1* cDNA: Cloning and sequencing

**Fig 1:**
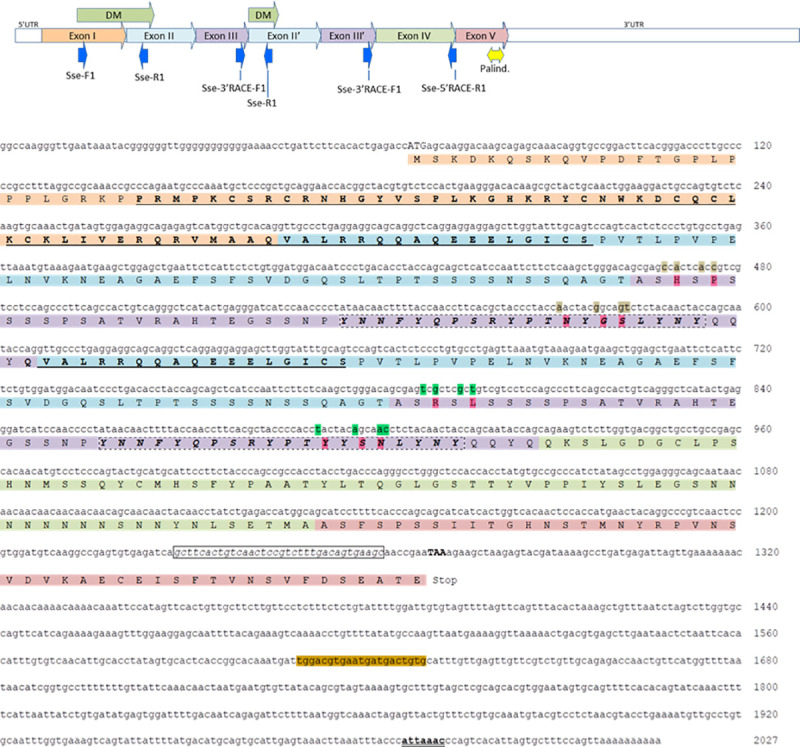
Structure of the cDNA *dmrt1* gene in *Solea senegalensis*. Upper figure: schematic illustration of exon structure, binding sites and orientation or primers. Exonic structure: Exon I–Exon II- Exon III–Exon II’–Exon III’–Exon IV–Exon V. Because of the exons duplication event, *primes* located in exon II and III also bind to exons II’ and III’. Lower figure: DM Domain is displayed underlined; palindromic sequence is bordered with black line before the TAA stop codon; polyadenylation signal with sequence ATTAAAC; in green 8 SNPs located in exons 3 and 3' can be observed and aminoacid changes are highlighted in red; finally orange sequence shows a miRNA sequence.

*Dmrt1* expression in male and female gonad samples was first checked using primers *Sse-F1* and *Sse-R1* (Table 1). The PCR products showed one or two bands in electrophoresis gel (S1 Fig). These PCR products were cloned and sequenced. With the amplified positive samples obtained, a 3’ RACE procedure was applied and again different size products were found. 3’ RACE PCR products were cloned and twenty of them sequenced, displaying several partial-length cDNA sequences. With the aim of securing overlapping sequences to obtain the complete *dmrt1* cDNA sequence, all the 3’UTR sequences were analyzed, and a 5’ RACE primer (*Sse-5' RACE-R1*, Table 1), localized at the 3’ end of the 3' RACE primer (Fig 1), was designed. After applying the 5’ RACE procedure to RNA from positive *dmrt1* expression samples, PCR products obtained in 5’ RACE were cloned and sequenced. The longest 5’ RACE sequence (5UTR_C6_H_SSH_W clone, 1135 bp long) overlapping the longest 3’ RACE product (3UTR_C9_H_SSH-W, 1139 bp long) from the same individual, was used to assemble the complete *dmrt1* cDNA from *S*. *senegalensis* (S1 Sequences) (a summary of sequenced clones can be found in S2 Table and S2 Fig). The complete *dmrt1* cDNA obtained is 2027 bp long.

The coordinates of each exon, 3’UTR, 5’UTR and other relevant elements, together with primers and palindromic sequences, were found by analysis using the BLAST algorithm. As shown in Fig 1, we were able to find an open-reading frame (ORF) of 1206 bp in length, a 3’UTR of 757 bp in length and 5’UTR of 66 bp in length.

Surprisingly, a duplication of exons 2 and 3 was observed in the *dmrt1* gene of *S*. *senegalensis* (Fig 1). The exon structure was as follows: exonI-exonII-exonIII-exonII’-exonIII’-exonIV-exonV. Localized at the end of exon V, seven nucleotides before the Stop codon “TAA”, a palindromic sequence of 34 bp long was also observed.

It should be noted that the primer *Sse-R1* was localized on exon II (Fig 1). Due to the exon duplication, it is also localized on exon II’. In the same way, the primer *Sse-III’* RACE-F1, localized in exon III, was also found on exon III’. Lastly, at the end of the 3’UTR sequence, a putative polyA-signal “ATTAAAC” was found.

The translation of *dmrt1* cDNA sequence showed that it encodes a predicted 402 aa protein (Accession number in progress). The exons were annotated and the presence of the DM domain was checked. As shown in Fig 1, the DM domain was localized on part of exon I and II. Due to the exon duplication event, part of the DM domain was found also on exon II’. A characteristic Y-rich domain was found on exon III and consequently on exon III’.

**Fig 2:**
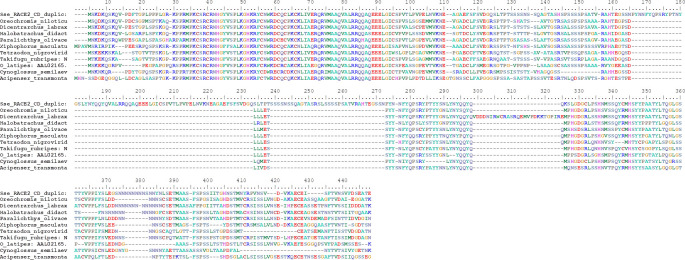
Alignment of amino acid sequences corresponding to the *dmrt1* gene from several Teleostei species. Accession numbers: *Oreochromis niloticus*: AAF79931.1; *Dicentrarchus labrax*: CAQ52796.1; *Halobatrachus didactylus*: AGN49325.1; *Paralichthys olivaceus*: ACD62474.1; *Xiphophorus maculatus*: AAN65377.1; *Tetraodon nigroviridis*: AAN74844.1; *Takifugu rubripes*: NP_001033038.1; *Oryzias latipes*: AAL02165.1; *Cynoglossus semilaevis*: ABS31368.1; *Acipenser transmontanus*: AAL18252.1.

When the *dmrt1* amino acid sequence of *S*. *senegalensis* was aligned with that of other fish species (Fig 2), the duplication was evident, and a large gap was observed in the duplicated region containing exons II-III and II’-III’.

**Fig 3:**
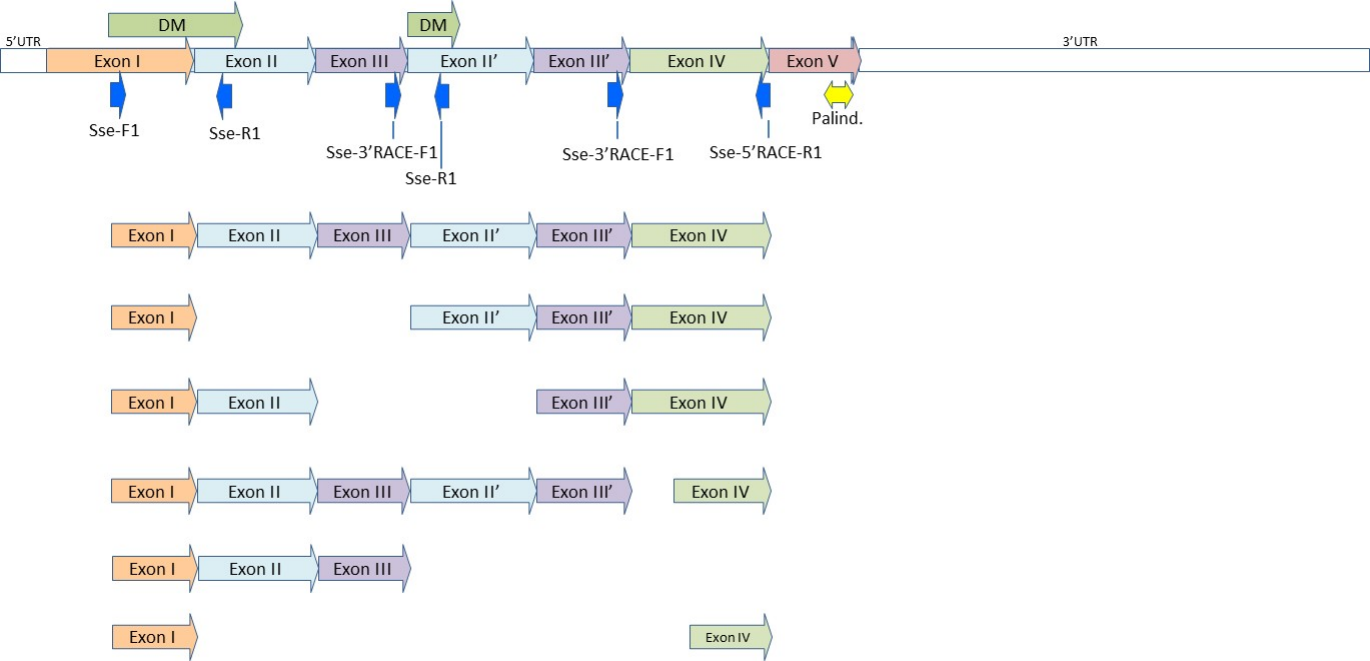
Alternative splicing in the *dmrt1* gene of *Solea senegalensis*. Six different transcripts have been observed when using *primers* located in exons I and IV.

Results after comparing exon sequences II-III and II’-III’ exon sequences (Fig 1), showed the complete identity between exons II and II’. However, in exon comparison III and III’, eight SNPs (single nucleotide polymorphisms) were observed. These SNPs were localized at the beginning and the end of these duplicated exons. These SNPs produce changes in the amino acid sequence: AS*H*S*P*S and AS*R*S*L*S at the beginning of exon III and exon III’, respectively; and T*N*Y*GS*L and T*Y*Y*SN*L at the end of exons III and III’, respectively (Fig 1). These polymorphisms allow exons III and III’ to be distinguished between each other. After finding the duplicated exons structure in the *dmrt1* gene of *S*. *senegalensis*, PCR reactions were carried out using *Sse-F1* and *Sse-5' RACE-R1* primers. These primers were localized in exon I and exon IV (end of this exon, Fig 1), so the duplicated regions could be amplified. PCR products showed different sizes and several of them, obtained from males and female gonads, differing in length, were cloned and sequenced (17 clones). The largest sequences (more than 900 bp), together with a long 5’RACE sequence (UTR_C6_H_SSH_W clone, Fig 3 and S2 Table), were aligned and analyzed. In all these sequences the exon duplication (the I-II-III-II’-III’- IV structure) was found.

Sequences of less than 900 bp in length (from exons I to IV) showed a range from 157 to 651 pb. When all the sequences from this region were analyzed, large polymorphisms were discovered. All the polymorphisms were produced by alternative splicing of exons (S2 Fig). Not one of these alternative isoforms was associated with sex nor origin of the fish. It was possible to observe various combinations of exons, such as: I-II’-III’-IV; I-II-III-II’-III’-IV; and I-II-III’-IV (Fig 3).

In addition to the study of this cDNA region extending from exon I to exon IV, with the primers previously mentioned, all clones sequenced in this study (BAC screening, expression test, RACE and cDNA fragment primers, Table 1) were studied to analyze the alternative splicing found in the *dmrt1* gene. The number of clones studied was 48(17 clones with *Sse-F1* and *Sse-5' RACE-R1* primers, and 31 from other combinations, S2 Table) from 5 males and 4 females. Five sequences obtained from one female from the 5’RACE sequences, showed four different exon combinations. Twenty sequences from the 3’RACE procedure, from 3 males and 2 females, showed 6 isoforms, and six sequences from the combination of primers *Sse-F1* and *Sse-R1* (anchored to exons I and II/II’ respectively) showed 3 isoforms (S2 Table and S2 Fig).

Twelve 3’UTRs from the 3’RACE sequences were aligned, and again polymorphisms were revealed. The results described two groups of sequences. The largest (10 sequences) has a length of 800 bp (approx.) and the shortest (2 sequences) of less than 400 bp. A high degree of homology was found in all aligned sequences. The shortest sequences were aligned at the end of long sequences (S2 Table and S2 Fig).

### *Dmrt1* interaction with other related proteins

For the purpose of searching for miRNA elements that could be acting as regulatory elements of *dmrt1* expression, the 3'UTR was used as a query in the mature miRNA sequences (filters by Teleostei) of the Mirbase database. The alignment of this 3’UTR sequence to mature miRNA produced a match (score: 78, identity and coverage: 100%, and e-value: 1.9) with the *ccr-miR-2192* mature miRNA, which is 21 bp in length; this element was localized at 336 bp from exon V.

**Fig 4:**
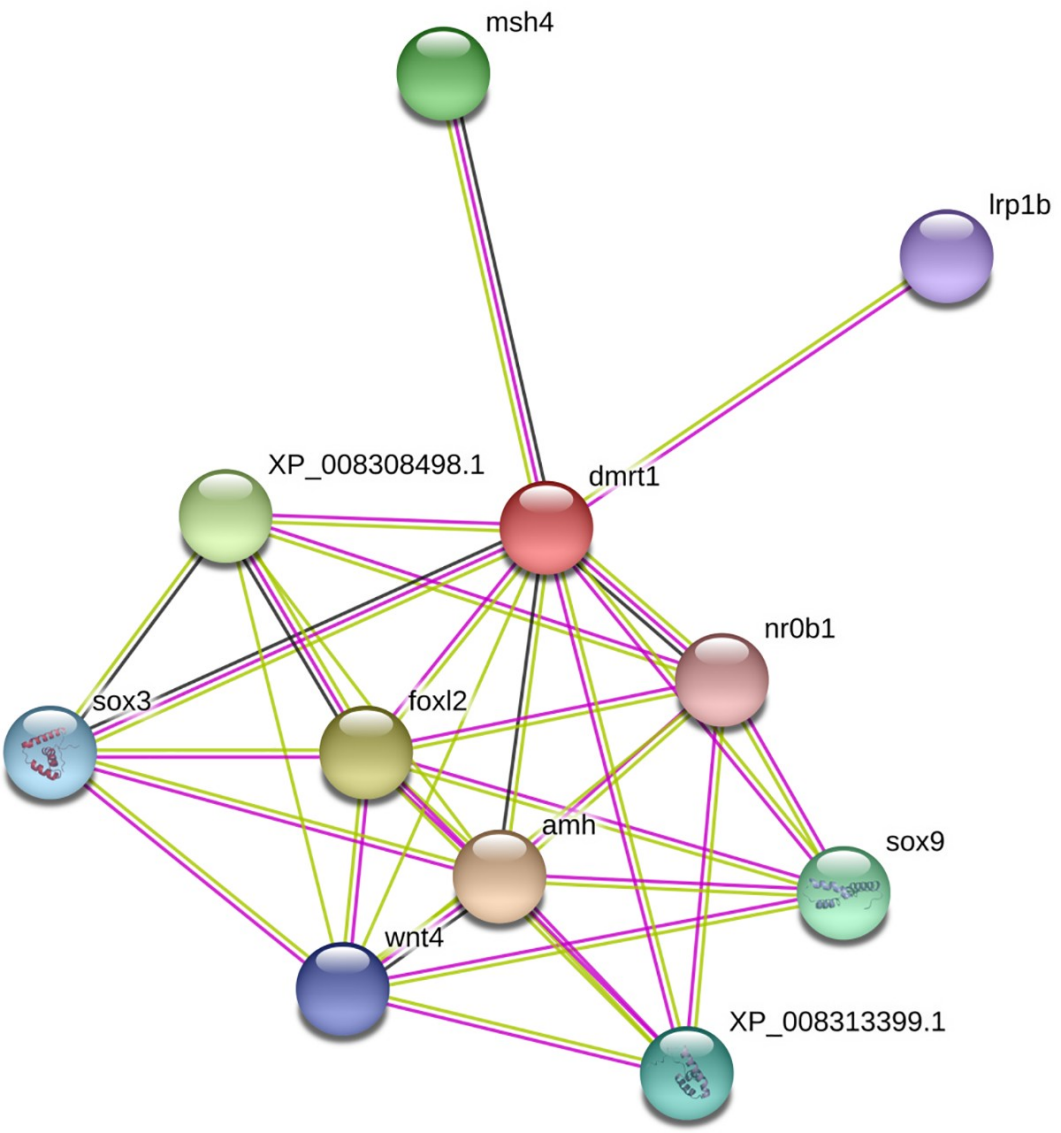
Interaction network between DMRT1 protein and other factors/ proteins, as predicted by STRING. The empty nodes mean proteins of unknown 3D structure and filled nodes some 3D structure is known or predicted for them. *sox3*: Transcription factor SOX-3; *nr0b1*: Nuclear receptor subfamily 0 group B member 1; *XP_008308498*.*1*: Aromatase-like; *sox9*: SRY (sex determining region Y); *box 9*: Transcription factor SOX-9; XP_008313399.*1*: Transcription factor SOX-9 isoform X1; *msh4*: mutS protein homolog 4; *foxl2*: Fork head box protein L2; *wnt4*: Protein Wnt-4; *amh*: Muellerian-inhibiting factor; *lrp1b*: Low-density lipoprotein receptor-related protein 1B; *dmrt1*: Doublesex- and mab-3-related transcription factor 1 isoform X1. Pink-line: experimentally determined; Green-line: gene neighborhood; Red-line: gene fusions; Blue-line: gene co-occurrence.

Protein-protein interacting (PPI) networks of the *dmrt1* gene of *S*. *senegalensis*, together with other proteins, were investigated using the STRING database (Fig 4). The results showed matches with *dmrt1* protein from *C*. *semilaevis* and a protein interaction network with 11 nodes (*dmrt1* plus 10 other proteins) and 32 edges, with an average node degree of 5.82, an average local clustering coefficient of 0.876, and a PPI enrichment *p-value* of 7.27e-08. As can be observed in Fig 4, the *dmrt1* protein interacted with *sox3* (transcription factor SOX-3), *nr0b1* (nuclear receptor subfamily 0 group B member 1), Aromatase-like, *sox9* (SRY-box 9; transcription factor SOX-9), transcription factor SOX-9 isoform X1, *msh4* (mutS protein homolog 4), *foxl2* (forkhead box protein L2), *wnt-4* (protein Wnt-4), *amh* (Muellerian-inhibiting factor) and *lrp1b* (low-density lipoprotein receptor-related protein 1B), with confidence levels of 0.783, 0.698, 0.903, 0.793, 0.793, 0.878, 0.910, 0.751, 0.918, and 0.709 respectively.

### Phylogenetic analysis

**Fig 5:**
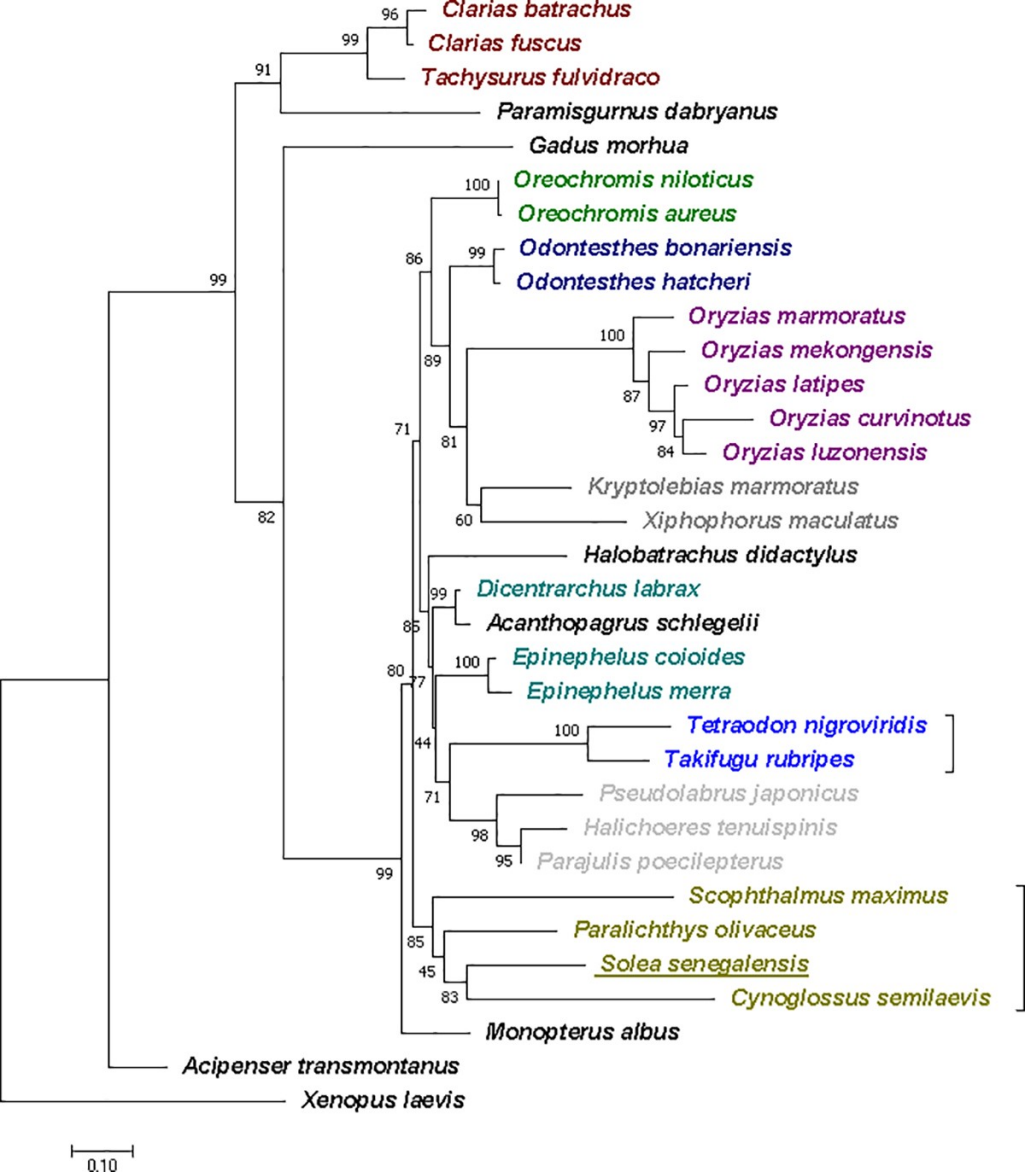
Phylogenetic tree showing the relationship between different *dmrt1* sequences. The bootstrap test is shown next to the branches. GenBank accession numbers: AAP84972.1, *Acanthopagrus schlegelii*; AAL18252.1, *Acipenser transmontanus*; ACR77514.1, *Clarias batrachus*; AEN92271.1, *Clarias fuscus*; ABS31368.1, *Cynoglossus semilaevis*; CAQ52796.1, *Dicentrarchus labrax*; ABK15558.1, *Epinephelus coioides*; ACD62373.1, *Epinephelus merra*; ACB97630.1, *Gadus morhua*; AAO18650.2, *Halichoeres tenuispinis*; AGN49325.1, *Halobatrachus didactylus*; ABG89135.1, *Kryptolebias marmoratus*; AAP80398.1, *Monopterus albus*; AAP84606.3, *Odontesthes bonariensis*; ACG69835.1, *Odontesthes hatcheri*; ABA29161.1, *Oreochromis aureus*; AAF79931.1, *Oreochromis niloticus*; BAC65995.1, *Oryzias curvinotus*; AAL02165.1, *Oryzias latipes*; AAS91465.1, *Oryzias luzonensis*; AAS91466.1, *Oryzias marmoratus*; AAS91464.1, *Oryzias mekongensis*; BAM62886.1, *Parajulis poecilepterus*; ACD62474.1, *Paralichthys olivaceus*; ABK88911.1, *Paramisgurnus dabryanus*; AAY64468.1, *Pseudolabrus japonicus*; AWP07629.1, *Scophthalmus maximus*; ADM07317.1, *Tachysurus fulvidraco*; NP_001033038.1, *Takifugu rubripes*; AAN74844.1, *Tetraodon nigroviridis*; NP_001089969.1, *Xenopus laevis*; AAN65377.1, *Xiphophorus maculatus*.

The phylogenetic tree constructed from the *dmrt1* amino acid sequence, showed that most of the fish species analyzed could be clustered in 3 well-supported main groups (Fig 5). One group contains the four Pleuronectiformes species studied here: *S*. *maximus*, *C*. *semilaevis*, *Paralichthys olivaceus* and *S*. *senegalensis*. Another group consists mainly of species from the Spariformes, Perciformes, Labriformes, Batrachoidiformes and Tetraodontiformes orders. The third main group comprises Cyprinodontiformes, Atheriformes, Cichliformes and Beloniformes orders. The other species are in branches out of the main group of fish species and belong to orders Acipenseriformes, Siluriformes, Gadiformes and Cypriniformes, and others. Sequences from the same genus were, in all cases, grouped in branches with high bootstrap values (Fig 5).

### *Dmrt1* gene structure and its association with repetitive elements

**Fig 6:**
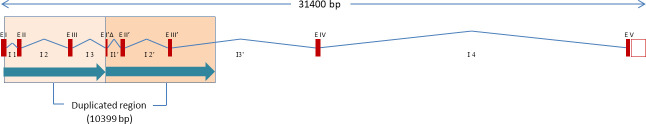
Schematic representation of *dmrt1* gene in the genome of *Solea senegalensis*. The gene is 31,4 kb long. It shows seven exons (with EII and EIII duplicated as EII’ and EIII’, EI’Δ is a partial and non-functional exon) and seven introns. The exons are indicated by red boxes and introns by blue lines. Empty boxes denote the 3’UTR region. The full duplicated region is 10,4 kb long. The region begins from the end of Exon I to the end of intron 3, and is next duplicated until part of intron 3’. The first region (light pink box) comprises end of Exon I–Intron 1 –Exon II- Intron 2—Exon III–Intron 3. The duplicated region comprises Exon I’Δ–Intron I’–Exon 2’- Intron II’—Exon III’–part of Intron 3’.

**Fig 7:**
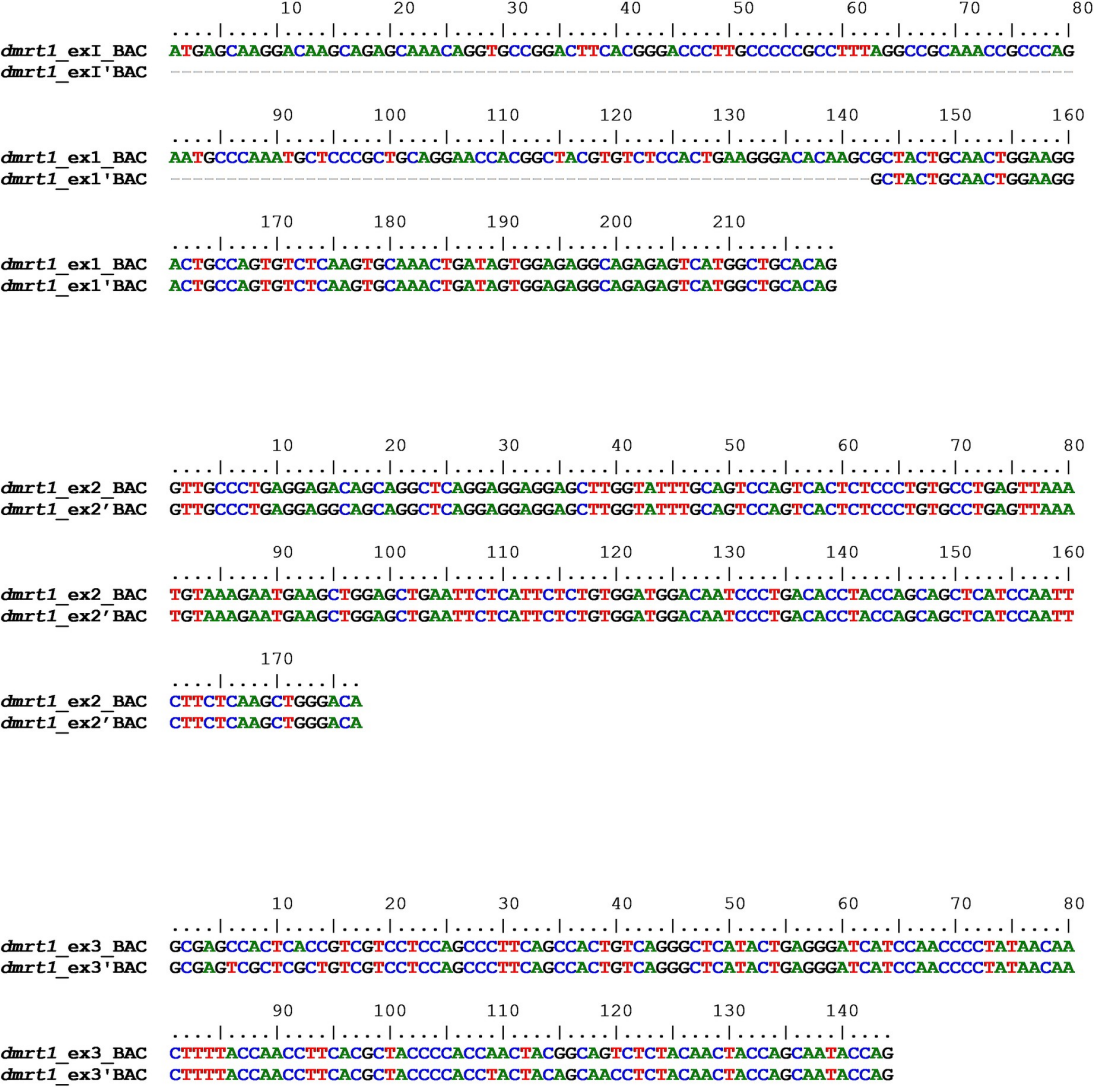
Alignment of duplicated exons of *dmrt1* gene from BAC sequence. Alignments: Exon I-Exon I’Δ; Exon II–Exon II’ and exon III–exon III’.

To investigate the structure of the *dmrt1* gene in the genome, a BAC sequence from clone 48K7 [32] was used. The *dmrt1* cDNA of *S*. *senegalensis* described in this paper was used in blast2seq search *vs* BAC sequences containing *dmrt1* gene. The results show a *dmrt1* gene 31400 bp long with an unexpected duplication, involving exons II and III, and a partial sequence of exon I (designated exon I’Δ from now on) between exon III and exon II’. The complete structure of the *dmrt1* gene in *S*. *senegalensis*, with the duplicated exons, is: “I-II-III-I’Δ -II’-III’-IV-V” (Fig 6). After aligning duplicated exons with each other, it was found that the exon I’Δ aligned in the second part of exon I (77/219 match, 35% coverage) with 100% identity (Fig 7). Analyzing the open-reading frame of this exon I’ Δ, in relation to codons described in the cDNA previously, an exon rupture is found, deduced from the absence of a correct ORF. Exons II and II’ showed almost exact homology between the two. When exons III and III’ were aligned, the eight SNPs previously observed in cDNA sequences were found, thus supporting this result (Fig 7).

To analyze the genome structure and the extent of the duplication in the *dmrt1* gene, several alignments were carried out between different regions of the sequences. As shown in Fig 6, the duplication begins exactly at exon I’Δ (between exon III and II’), and it extends to intron 6. Therefore, the sequence from exon I to the end of intron 3 (5081 bp) is duplicated following the sequence (5318 bp), with the duplication extending to 2107 bp of intron 6.

The length of the *dmrt1* gene in all forty-five of the fish species sequenced that are available in the ENSEMBLE database was studied and compared with the results for *S*. *senegalensis* (S4 and S5 Figs and S3 Table). The size of this gene, measured from transcripts with the maximum number of exons for each species, ranged from 1.81 kb (4 exons) in *Paramormyrops kingsleyae* to 28.43 kb (8 exons) in guppy. In the Pleuronectiformes order, only the tongue sole (*C*. *semilaevis*) and the turbot (*S*. *maximus*) are fully sequenced, and these present maximum values of 12.58 and 25.27 kb (6 and 5 exons respectively) in tongue sole, and 13.15 (6 exons) in turbot. No exon duplications were observed in any of the species analyzed.

The 143 transcripts from the forty-five fish species sequenced were then analyzed (S5 Fig). The lowest number of exons transcribed in fish was in *D*. *rerio* (2 *transcribed exons)* and the highest was 8 exons in guppy (*Poecilia reticulata*). Considering only the maximum number of exons transcribed by species, this value ranged from 4 (*P*. *kingsleyae* and western mosquitofish, *Gambusia affinis*) to 8 in guppy, displaying a normal distribution with a mean value of 5.38 (S5 Fig). The number of different transcripts for fish species analyzed showed that more than 65% of the species presented one to three different transcripts, and 35% presented between four and ten (S3 Table).

Introns 1, 2 and 3 (between exons I-II, II-III and III-I’partial respectively) were compared with introns 1’, 2’ and the overlapping sequence of intron 3’ (between exons I’-II’, II’-III’ and part of III’ respectively) (S6 Fig). The analysis of polymorphisms in intron 1 and 4 revealed 118 sites with gaps and 577 sites without gaps. These un-gapped sites comprised 566 monomorphic and 11 polymorphic sites with a Jukes and Cantor (Pi-JC) nucleotide diversity value of 0.019. Introns 2 and 5 showed a total number of sites (excluding gaps) of 2006, comprising 1674 monomorphic and 332 polymorphic sites, and 395 gapped sites. The analysis of DNA polymorphisms found a Pi-JC nucleotide diversity of 0.187. Finally, after comparison between intron 3 and the homologous part of intron 6, 874 gapped sites out of 2328, 1341 invariable sites and 113 polymorphic nucleotides were found. From 1454 un-gapped sites, the Pi-JC nucleotide diversity was 0.082 (Table 2).

**Fig 8:**
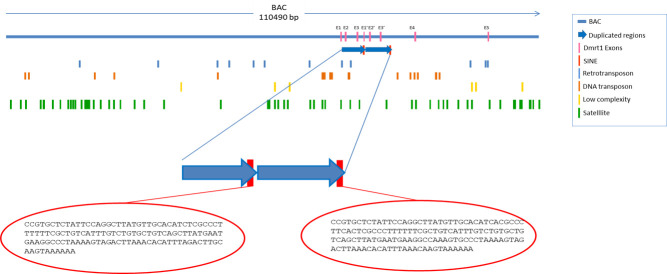
Distribution of repetitive elements in a BAC clon *of S*. *senegalensis*, coming from a genome library, containing the *dmrt1* gene. Green boxes are satellite elements. Yellow boxes are low complexity sequences. Orange boxes are DNA transposons. Blue boxes are retrotransposons. Red boxes are flanking duplicated regions with SINE element obtained from NGS BAC reads. Pink boxes are *dmrt1* exons. Duplicated regions are displayed as blue arrows and DNA BAC as a blue line.

The analysis made of the presence and distribution, in BACs containing the *dmrt1* gene, of repetitive elements such as DNA satellite and Transposable elements (TEs), is described in Fig 8.

Different DNA satellites and low complexity regions are scattered throughout the BAC sequence. DNA transposons and retrotransposons are also widely distributed throughout the BAC. The presence of a SINE element should be noted, because it is located right at the end of the second repeated region in the *dmrt1* gene (Fig 8, S7 Fig).

## Discussion

The *dmrt1* cDNA obtained in *S*. *senegalensis* is 2027 bp long, and from this an ORF coding for a protein of 422 aa is obtained. The characteristics and size of the cDNA sequences of the *dmrt1* gene of other fish species have been reported previously, as follows: cDNA length of 922 bp, and ORF of 744 bp, for a protein of 247 aa in *C*. *alburnus* [36]; cDNA length 6415 bp and an ORF 900 bp, for a protein of 298 aa in *M*. *salmoides* [37]; in Nile tilapia (*O*. *nitolicus*), a *dmrt1* gene with a length of 1471 bp has been detected with an ORF of 1118 bp, which codes for a protein of 372 aa [38]; in zebrafish (*Dario rerio*), the total length of *dmrt1* is 1390 bp, which codes for a protein of 267 aa [18]; and the cDNA of *dmrt1* has a length of 1855 bp, with an ORF of 864 bp, which codes for a protein of 287 aa in *Astyanax altiparanae* [39].

It should be noted that, with respect to the species cited above, the ORF of *S*. *senegalensis* is between 13% and 70% larger (13% more than in *O*. *nitolicus*; 41% more than in *M*. *salmoides*; 47% more than in *Astyanax altiparanae*; 58% more than in *Dario rerio*; and 70% more than in *C*. *alburnus*); this may be explained by the appearance of a duplication of exons II and III, which also includes a partial duplication of the DM region.

The ORF of the *dmrt1* gene of *S*. *senegalensis* showed a highly conserved DM domain (from amino acid 26 to 90: a total of 64 amino acids). This domain shares the same typical zinc-finger structure as other members of the *dmrt* family, i.e. two cysteines (Cys) at positions 31 and 34 of the amino acid sequence, histidine (His) at positions 37 and 46, and Cys at positions 50, 55, 57 and 60, which form a "C2H2C4" zinc-finger model by binding with zinc ions (Figs 4 and 5).

The "C2H2C4" structure in the *dmrt1* DM domain of *S*. *senegalensis* is the same as that of *Micropterus salmoides* [37], and that of Hong Kong catfish (*Clarias fuscus*) and yellow catfish [40], and shares the same position among the corresponding amino acids. The only difference is that the "C2H2C4" structure in *M*. *salmoides* and *S*. *senegalensis* begins at amino acid 31, while that of Hong Kong catfish and yellow catfish begins at 33. *S*. *senegalensis* has the same location of the amino acids corresponding to the zinc-finger structure in the DM domain as other Perciform fish species, which begin at amino acid 31.

The results of Protein-protein interacting networks of *S*. *senegalensis* showed matches with *dmrt1* protein from *C*. *semilaevis* and a protein interaction network with 11 nodes in which proteins related to sex determination/differentiation are located. That gives a hint about its interaction with transcription factors and participation in the sex differentiation of teleost fish.

In the phylogenetic tree relationship of the *dmrt1* gene in *S*. *senegalensis* is consistent with the evolutionary position of its species. We observe that the sequence of the protein *dmrt1* of *S*. *senegalensis* was the closest in evolution to the sequences of *S*. *maximus*, *C*. *semilaevis*, and *P*. *olivaceus*, all flatfish of the Pleuronectiformes order. This result contrasts with that in the study conducted by [29] using 10 concatenated protein sequences of sex-related genes (*amh*, *cyp19a1a*, *dmrt2*, *dmrt3*, *dmrt4*, *lhb*, *nanos3*, *sox3*, *sox6*, *vasa)*. In this phylogenetic analysis *S*. *senegalensis i*s phylogenetically close to *O*. *latipes*, a species with an XX/XY system and the *dmrt1* as the sex master gene.

The miRNA (*ccr-miR-2192*) found in exon V of *dmrt1* has been reported as a miRNA that is differentially expressed in the developmental process of common carp skeletal muscle [41]. MiRNAs regulate the expression of genes that are important in establishing sexual dimorphic traits in animals [42].

To date, only two studies have explored miRNA sequences in *S*. *senegalensis*; one study evaluated the role of miRNAs in thermal growth plasticity [43] and the other more recent study identified miRNAs from sequences of a BAC library by integrating sequence information with data on physical chromosome location [44].

The structure of the *dmrt1* genomic DNA of *S*. *senegalensis* showed an unexpected duplication of exons II and III. Although we have not found exon duplications in the *dmrt1* gene in the forty-five fish species sequenced so far (ENSEMBLE, data base), according to Peng and Li [45] (2009) 8% of the genes of *H*. *sapiens*, and 7% of mouse genes containing duplicated exons.

The importance of gene duplication in the formation of new genes has been recognized for decades. Tandem duplication of a genomic segment is the most common way to produce redundant genes. This duplication of a genomic segment can occur on a smaller scale resulting in duplicate exons, rather than complete genes. Compared to the gradual and minor functional divergence between complete duplicate genes, internal duplication of genes can lead to immediate acquisition of new function and sometimes provide a substantial selective advantage. This type of tandem exon duplication is common in genomes and is an important mechanism for expanding gene function [46].

Several studies carried out on fully-sequenced human genomes reveal that internal duplications of genetic segments occur with a high frequency (0.001–0.013 duplications/gene per million years), similar to that of complete genetic duplications, so that 8–17% of the genes in a genome carry duplicated intron and/or exon regions [47].

Moreover, many exon duplication events leading to alternative processing have been described. One possible way for a gene transcript with two repeated exons to achieve functional redundancy is by mutually-exclusive processing, a special type of alternative processing. Alternative processing expands proteomic diversity and regulates developmental and tissue-specific biological processes [48].

There are several implications for multiple processing in gonadogenesis. First, the various isoforms of *dmrt1* generated by alternative processing can provide potentially diverse targets for different factors interacting upstream and downstream in sexual regulation. Few DM factor regulation targets have been identified to date, and most data are from *Drosophila dsx* and *C*. *elegans MAB-3* [49]. In *Drosophila*, the *dsx* gene is considered to be the last sex regulation gene because its proteins bind to and directly regulate the transcription of two of the genes encoding terminal sex differentiation proteins, the *Yolk-1* and *Yolk-2* protein genes [50].

The abundance of transcripts detected at the gene level has led to the assumption that, if these are translated into proteins, they could be playing a determining role in the complexity of mammals [51].

The analysis of the exon-exon junctions of *S*. *senegalensis* cDNA splice study shows that the splice sites are localized at the end of each exon, the -AG- sequence, in all the splices; this finding coincides with those of several studies, including those carried out on zebrafish, comparing *dmrt1* with that of medaka, Takifugu, mouse and human. Aligning the intron-exon junctions of *dmrt1* of these diverse species showed that the splice sites (GT-AG) in all these intron-exons of *dmrt1* are conserved in both mammals and fish [19]. When we applied a distribution analysis of the nucleotide diversity found among introns, we also observed that this diversity was less in the environment of the splice sites (S6 Fig).

Exon shuffling or random combination of exons is a molecular mechanism for the formation of new genes, and RNA transposons have been reported to control exon shuffling in some genes [52].

The hypothesis that best explains the duplication of exons found in *S*. *senegalensis* is the exon-shuffling mechanism mediated by a SINE transposer, the presence of which is significant, since it is found at the end of the second region repeated in the *dmrt1* gene (Fig 8). SINEs are retrotransposons that have had notable reproductive success during the course of mammalian evolution and have played an important role in shaping mammalian genomes. A study of canine genomes has shown that, when transcribed in antisense orientation, they provide splice acceptance sites that can result in the incorporation of new exons [53].

The functional and evolutive meaning of the intragenic duplication and the high variability of mRNA isoforms found in *S*. *senegalensis* has to be further elucidated.

## Conclusion

The genomic structure of the *dmrt1* of *S*. *senegalensis* reveals a gene of 31400 bp composed of 7 exons and 6 introns. It contains an unexpected duplication of more than 10399 bp, involving part of exon I, exons II and III, and a SINE element found in the sequence that is proposed as the factor responsible for the duplication. The molecular characterization of this gene will undoubtedly enhance its functional analysis and hence the understanding of how sex develops in *S*. *senegalensis* and other flatfish.

## Supporting information

S1 Sequences cDNA sequences obtained from isolated clones from expressed *S. senegalensis dmrt1* gene. Forty eigth sequences were used to study the cDNA structure of *dmrt1* gene of *S*. *senegalensis*. A consensus sequence is also shown.
(TXT)

S1 Table Accession numbers and taxonomy of *dmrt1* protein sequences used in the phylogenetic analysis. Thirty two sequences were used to carry out the phylogenetic tree of dmrt1 gene of *S*. *senegalensis*.
(DOCX)

S2 Table Summary of *dmrt1* cDNA gene clones in *Solea sengalensis*. Clone and sample Ids, animal sex, sequence length (bp), purpose in the study and patterns are shown. Expression test: primers used to test *dmrt1* expression in RNA extracted from gonads; BAC screen: Primers used to amplify sequences from the BAC containing the *dmrt1* gene.
(DOCX)

S3 Table Number of *dmrt1* transcripts per 45 fish species obtained after data mining of ENSEMBLE database. A total of 143 transcripts were obtained from 45 fish species ranging from 1 to 10 in the species.
(DOCX)

S1 Fig Electrophoresis gel showing two amplification products after PCR using primers located in the cDNA duplicated region of the *S. senegalensis dmrt1* gene. Lane 1: Hypperladder 2 molecular weight marker (EcogenTM); Lane 2 and 3 PCR amplicons after using Sse-F1 and Sse-R1 primers.
(DOCX)

S2 Fig Length of *dmrt1* clone sequences in *S. senegalensis*. Forty eight cDNA sequences of *dmrt1* from males and females of *S*. *senegalensis* are shown.
(DOCX)

S3 Fig Alternative splicing in the *dmrt1* gene of *Solea senegalensis*. Several different transcripts have been observed when using *primers* located in exons I, II, III (and therefore II’ and III’ as coming from the duplication event), and exon IV.
(DOCX)

S4 Fig Size of *dmrt1* gene in 45 fish species. Several transcripts, with alternative exon splicing, are described from every species in Ensemble data base. (A) The gene sizes (kb) considering the sequence length comprising from 5’UTR to 3’UTR are displayed. (B) Basic statistics such as mean, median, mode, std. deviation, variance, range, minimum, maximum, percentiles are shown. (C) Box and whisker plot are displayed. Fish species: Amazon molly (*Poecilia formosa*), Asian bonytongue (*Scleropages formosus*), Bicolor damselfish (*Stegastes partitus*), Channel catfish (*Ictalurus punctatus*), Climbing perch (*Anabas testudineus*), Cod (*Gadus morhua*), Eastern happy (*Astatotilapia calliptera*), Fugu (*Takifugu rubripes*), Greater amberjack (*Seriola dumerili*), Indian medaka (*Oryzias melastigma*), Mangrove rivulus (*Kryptolebias marmoratus*), Mexican tetra (*Astyanax mexicanus*), Midas cichlid (*Amphilophus citrinellus*), Paramormyrops kingsleyae (*Paramormyrops kingsleyae*), Platyfish (*Xiphophorus maculatus*), Red-bellied piranha (*Pygocentrus nattereri*), Sailfin molly (*Poecilia latipinna*), Sheepshead minnow (*Cyprinodon variegatus*), Spiny chromis (*Acanthochromis polyacanthus*), Spotted gar (*Lepisosteus oculatus*), Tiger tail seahorse (*Hippocampus comes*), Tilapia (*Oreochromis niloticus*), Western mosquitofish (*Gambusia affinis*), Zig-zag eel (*Mastacembelus armatus*), Clown anemonefish (*Amphiprion ocellaris*), Shortfin molly (*Poecilia mexicana*), Stickleback (*Gasterosteus aculeatus*), Tetraodon (*Tetraodon nigroviridis*), Zebra mbuna (*Maylandia zebra*), Swamp eel (*Monopterus albus*), Tongue sole (*Cynoglossus semilaevis*), Turbot (*Scophthalmus maximus*), Japanese medaka HdrR (*Oryzias latipes*), Japanese medaka HNI (*Oryzias latipes*), Mummichog (*Fundulus heteroclitus*), Ballan wrasse (*Labrus bergylta*), Makobe Island cichlid (*Pundamilia nyererei*), Burton's mouthbrooder (*Haplochromis burtoni*), Northern pike (*Esox lucius*), Zebrafish (*Danio rerio*), Guppy (*Poecilia reticulata*), Lyretail cichlid (*Neolamprologus brichardi*), Orange clownfish (*Amphiprion percula*), Periophthalmus magnuspinnatus (*Periophthalmus magnuspinnatus*), Yellowtail amberjack (*Seriola lalandi dorsalis*).
(DOCX)

S5 Fig Number of exons of *dmrt1* gene in fish species. (A) Several transcripts, with alternative exon splicing, are described from every species in Ensemble database. (B) Histogram and normal curve are displayed showing some statistics as mean (5,38), Std. Deviation (1,073) from 143 data obtained of 45 fish species. Fish species: Amazon molly (*Poecilia formosa*), Asian bonytongue (*Scleropages formosus*), Bicolor damselfish (*Stegastes partitus*), Channel catfish (*Ictalurus punctatus*), Climbing perch (*Anabas testudineus*), Cod (*Gadus morhua*), Eastern happy (*Astatotilapia calliptera*), Fugu (*Takifugu rubripes*), Greater amberjack (*Seriola dumerili*), Indian medaka (*Oryzias melastigma*), Mangrove rivulus (*Kryptolebias marmoratus*), Mexican tetra (*Astyanax mexicanus*), Midas cichlid (*Amphilophus citrinellus*), Paramormyrops kingsleyae (*Paramormyrops kingsleyae*), Platyfish (*Xiphophorus maculatus*), Red-bellied piranha (*Pygocentrus nattereri*), Sailfin molly (*Poecilia latipinna*), Sheepshead minnow (*Cyprinodon variegatus*), Spiny chromis (*Acanthochromis polyacanthus*), Spotted gar (*Lepisosteus oculatus*), Tiger tail seahorse (*Hippocampus comes*), Tilapia (*Oreochromis niloticus*), Western mosquitofish (*Gambusia affinis*), Zig-zag eel (*Mastacembelus armatus*), Clown anemonefish (*Amphiprion ocellaris*), Shortfin molly (*Poecilia mexicana*), Stickleback (*Gasterosteus aculeatus*), Tetraodon (*Tetraodon nigroviridis*), Zebra mbuna (*Maylandia zebra*), Swamp eel (*Monopterus albus*), Tongue sole (*Cynoglossus semilaevis*), Turbot (*Scophthalmus maximus*), Japanese medaka HdrR (*Oryzias latipes*), Japanese medaka HNI (*Oryzias latipes*), Mummichog (*Fundulus heteroclitus*), Ballan wrasse (*Labrus bergylta*), Makobe Island cichlid (*Pundamilia nyererei*), Burton's mouthbrooder (*Haplochromis burtoni*), Northern pike (*Esox lucius*), Zebrafish (*Danio rerio*), Guppy (*Poecilia reticulata*), Lyretail cichlid (*Neolamprologus brichardi*), Orange clownfish (*Amphiprion percula*), Periophthalmus magnuspinnatus (*Periophthalmus magnuspinnatus*), Yellowtail amberjack (*Seriola lalandi dorsalis*).
(DOCX)

S6 Fig Duplicated intron alignments and plots of entropy as a measure of probability of positional homology in *dmrt1* gene of *S. senegalensis*. (A) Introns 1 and 1’ alignment and their plot of entropy; (B) Introns 2 and 2’ alignment and their plot of entropy; (C) Intron 3 and its duplicated region in intron 3’ alignment and their plot of entropy.
(DOCX)

S7 Fig Analysis or transposable elements in *S. senegalensis dmrt1* gene. Upper figure shows the sequence of a cluster obtained after Graph-based clustering and characterization of repetitive sequences from 454 Roche NGS data of *Solea senegalensius* BAC clon 48K7. SINE sequence (SINE2-1B_DR) obtained after cluster BLAST search in Dfam database, is displayed in bold and underlined. Lower figure displays graph layout derived from a read cluster indicative of *S*. *senegalensis* repetitive sequence is displayed: Single reads are represented by nodes and their sequence overlaps by edges. This SINE element is located flanking the duplicated region in *dmrt1* gene.
(DOCX)
