# Supplementary material for: The genomic structure of the highly-conserved dmrt1 gene in Solea senegalensis (Kaup, 1868) shows an unexpected intragenic duplication
Source: PLoS One. 2020 Nov 2;15(11):e0241518. doi: 10.1371/journal.pone.0241518 (PMC7605655; doi:10.1371/journal.pone.0241518)
Supplement: S1 Table — Thirty two sequences were used to carry out the phylogenetic tree of dmrt1 gene of S. senegalensis. (DOCX) [file pone.0241518.s002.docx]

**S1 Table:** **Accession numbers and taxonomy of *dmrt1* protein sequences used in the phylogenetic analysis.** Thirty two sequences were used to carry out the phylogenetic tree of dmrt1 gene of *S. senegalensis*.

| Acc. Number | Species | Order | Family |
| --- | --- | --- | --- |
| AAP84972.1 | *Acanthopagrus schlegelii* | Spariformes | Sparidae |
| AAL18252.1 | *Acipenser transmontanus* | Acipenseriformes | Acipenseridae |
| ACR77514.1 | *Clarias batrachus* | Siluriformes | Clariidae |
| AEN92271.1 | *Clarias fuscus* | Siluriformes | Clariidae |
| ABS31368.1 | *Cynoglossus semilaevis* | Pleuronectiformes | Cynoglossidae |
| CAQ52796.1 | *Dicentrarchus labrax* | Perciformes | Monoidae |
| ABK15558.1 | *Epinephelus coioides* | Perciformes | Serranidae |
| ACD62373.1 | *Epinephelus merra* | Perciformes | Serranidae |
| ACB97630.1 | *Gadus morhua* | Gadiformes | Gadidae |
| AAO18650.2 | *Halichoeres tenuispinis* | Labriformes | Labridae |
| AGN49325.1 | *Halobatrachus didactylus* | Batrachoidiformes | Batrachoididae |
| ABG89135.1 | *Kryptolebias marmoratus* | Cyprinidontiformes | Rivulidae |
| AAP80398.1 | *Monopterus albus* | Synbranchiformes | Synbranchidae |
| AAP84606.3 | *Odontesthes bonariensis* | Atheriniformes | Atherinopsidae |
| ACG69835.1 | *Odontesthes hatcheri* | Atheriformes | Atherinopsidae |
| ABA29161.1 | *Oreochromis aureus* | Cichliformes | Cichlidae |
| AAF79931.1 | *Oreochromis niloticus* | Cichliformes | Cichlidae |
| BAC65995.1 | *Oryzias curvinotus* | Beloniformes | Adrianichthyidae |
| AAL02165.1 | *Oryzias latipes* | Beloniformes | Adrianichthyidae |
| AAS91465.1 | *Oryzias luzonensis* | Beloniformes | Adrianichthyidae |
| AAS91466.1 | *Oryzias marmoratus* | Beloniformes | Adrianichthyidae |
| AAS91464.1 | *Oryzias mekongensis* | Beloniformes | Adrianichthyidae |
| BAM62886.1 | *Parajulis poecilepterus* | Labriformes | Labridae |
| ACD62474.1 | *Paralichthys olivaceus* | Pleuronectiformes | Paralichthyidae |
| ABK88911.1 | *Paramisgurnus dabryanus* | Cypriniformes | Cobitidae |
| AAY64468.1 | *Pseudolabrus japonicus* | Labriformes | Labridae |
| AWP07629.1 | *Scophthalmus maximus* | Pleuronectiformes | Scophthalmidae |
| ADM07317.1 | *Tachysurus fulvidraco* | Siluriformes | Bagridae |
| NP_001033038.1 | *Takifugu rubripes* | Tetraodontiformes | Tetraodontidae |
| AAN74844.1 | *Tetraodon nigroviridis* | Tetraodontiformes | Tetraodontidae |
| NP_001089969.1 | *Xenopus laevis* | Anura | Pipidae |
| AAN65377.1 | *Xiphophorus maculatus* | Cyprinodontiformes | Poeciliidae |
