# Supplementary material for: The genomic structure of the highly-conserved dmrt1 gene in Solea senegalensis (Kaup, 1868) shows an unexpected intragenic duplication
Source: PLoS One. 2020 Nov 2;15(11):e0241518. doi: 10.1371/journal.pone.0241518 (PMC7605655; doi:10.1371/journal.pone.0241518)
Supplement: S2 Table — Clone and sample Ids, animal sex, sequence length (bp), purpose in the study and patterns are shown. Expression test: primers used to test dmrt1 expression in RNA extracted from gonads; BAC screen: Primers used to amplify sequences from the BAC containing the dmrt1 gene. (DOCX) [file pone.0241518.s003.docx]

**S2 Table:** **Summary of *dmrt1* cDNA gene clones in *Solea sengalensis***. Clon and sample Ids, animal sex, sequence length (bp), purpose in the study and patterns are shown. Expression test: primers used to test *dmrt1* expression in RNA extracted from gonads; BAC screen: Primers used to amplify sequences from the BAC containing the *dmrt1* gene

| #clon_ID | Sex | length (bp) | Purpose | Pattern |
| --- | --- | --- | --- | --- |
| *3UTR_7_H_SSH-W Lote 4 Fwd 1127* | Female | 1127 | 3'RACE | NA-NA-0-0-3'-4-5-3UTR |
| *3UTR_C10_H_SSH-W Lote 4 Rev 389* | Female | 389 | 3'RACE | NA-NA-0-0-3'-4-5- |
| *3UTR_C1_H_SSH-W Lote 4 Rev 1138* | Female | 1138 | 3'RACE | NA-NA-0-0-3'-4-5-3UTR |
| *3UTR_C3_H_SSH-W Lote 4 Rev 1129* | Female | 1129 | 3'RACE | NA-NA-0-0-3'-4-5-3UTR |
| *3UTR_C4_M_SSM-W Lote 4 Rev 419* | Male | 419 | 3'RACE | NA-NA-0-0-3'-4-5- |
| *3UTR_C6_H_SSH-W Lote 4 Rev 696* | Female | 696 | 3'RACE | NA-NA-0-2'-3'-0-0-3UTR-partial |
| *3UTR_C8_H_SSH-W Lote 4 Rev 381* | Female | 381 | 3'RACE | NA-NA-0-0-3'-4'partial-0-3UTR-partial |
| *3UTR_C9_H_SSH-W Lote 4 Fwd 1139* | Female | 1139 | 3'RACE | NA-NA-0-0-3'-4-5-3UTR |
| *3UTR_c10_M_SSM-2 Fwd Lote 3 649bp* | Male | 649 | 3'RACE | NA-NA-0-2'-3'-0-0-3UTR-partial |
| *3UTR_c10_M_SSM-B Fw: Lote 1_436bp* | Male | 436 | 3'RACE | NA-NA-0-0-3'-4-5- |
| *3UTR_c3_M_SSM-2 Rv Lote 3 1335bp* | Male | 1335 | 3'RACE | NA-NA-0-0-3'-4-5-3UTR |
| *3UTR_c3_M_SSM-B Fw: Lote 1_379bp* | Male | 379 | 3'RACE | NA-NA-0-2'-3'partial-4-5- |
| *3UTR_c4_M_SSM-B Fw: Lote 1_397bp* | Male | 397 | 3'RACE | NA-NA-0-0-3'-4-5- |
| *3UTR_c5_M_-SSM-B Fw: Lote 1_1211bp* | Male | 1211 | 3'RACE | NA-NA-0-0-3'-4-5-3UTR |
| *3UTR_c5_M_SSM-2 Fwd Lote 3 1352bp* | Male | 1352 | 3'RACE | NA-NA-0-0-3'-4-5-3UTR |
| *3UTR_c6_M_SSM-2 Rv Lote 3 397bp* | Male | 397 | 3'RACE | NA-NA-0-0-3'-4-5- |
| *3UTR_c6_M_SSM-B Fw: Lote 1_1375bp* | Male | 1375 | 3'RACE | NA-NA-0-0-3'-4-5-3UTR |
| *3UTR_c7_M_SSM-2 Rev Lote 3 509bp* | Male | 509 | 3'RACE | NA-NA-0-0-3'-4-5- |
| *3UTR_c8_H_SSH-A Fw: Lote 1_1371bp* | Female | 1371 | 3'RACE | NA-NA-0-0-3'-4-5-3UTR |
| *3UTR_c8_M_SSM-2 Rev Lote 3 1347bp* | Male | 1347 | 3'RACE | NA-NA-3-2'-3'partial-4-5-3UTR |
| *5UTR_C10_H_SSH-W Lote 5 Fwd 152* | Female | 152 | 5'RACE | NA-NA-0-0-0-4-0-NA |
| *5UTR_C4_H_SSH-W Lote 5 Rev 365* | Female | 365 | 5'RACE | 0-0-0-0-0-4-5-NA |
| *5UTR_C5_H_SSH-W Lote 5 Fwd 78* | Female | 78 | 5'RACE | 0-0-0-0-0-4-NA-NA |
| *5UTR_C6_H_SSH-W Lote 5 Rev 1135* | Female | 1135 | 5'RACE | 1-2-3-2'-3'-4-NA-NA |
| *5UTR_C9_H_SSH-W Lote 5 Fwd 148* | Female | 148 | 5'RACE | NA-NA-0-0-0-4-0-NA |
| *Dmrt_c1_H_SSH-3 Fwd Lote 3 648bp* | Female | 648 | BAC_screen; Expression test | 0-2-3-NA-NA-NA-NA-NA |
| *Dmrt_c1_M_SSM-2 Rv Lote 3 175bp* | Male | 175 | BAC_screen; Expression test | 1-0-3partial-NA-NA-NA-NA-NA |
| *Dmrt_c4_M_SSM-2 Rv Lote 3 175bp* | Male | 175 | BAC_screen; Expression test | 1-0-3partial-NA-NA-NA-NA-NA |
| *Dmrt_c5_H_SSH-3 Fwd Lote 3 496bp* | Female | 496 | BAC_screen; Expression test | 1-2-3-NA-NA-NA-NA-NA |
| *Dmrt_c5_M_SSM-2 Fwd Lote 3 496bp* | Male | 496 | BAC_screen; Expression test | 1-2-3-NA-NA-NA-NA-NA |
| *Dmrt_c8_M_SSM-2 Fwd Lote 3 496bp* | Male | 496 | BAC_screen; Expression test | 1-2-3-NA-NA-NA-NA-NA |
| *SSH-16_163: femalePCR4 20_04-2018_163bp* | Female | 163 | exons I- IV cDNA analysis | 1-0-0-0-0-4-NA-NA |
| *SSH-16_648: femalePCR4 20_04_2018_648bp* | Female | 648 | exons I- IV cDNA analysis | 1-2-3-2'-3'-4-NA-NA |
| *SSH-16_969: femalePCR4 20_04_2018_969bp* | Female | 969 | exons I- IV cDNA analysis | 1-2-3-2'-3'-4-NA-NA |
| *SSH-3_428: femalePCR4 20_04_2018_428bp* | Female | 428 | exons I- IV cDNA analysis | 1-2-3-0-0-0-NA-NA |
| *SSH-3_651: femalePCR4 20_04_2018_651 bp* | Female | 651 | exons I- IV cDNA analysis | 1-0-0-2'-3'-4-NA-NA |
| *SSH-A_132: femalePCR4 20_04_2018_132bp* | Female | 132 | exons I- IV cDNA analysis | 1partial-0-0-0-0-4partial-NA-NA |
| *SSH-A_651: femalePCR4 20_04_2018_651bp* | Female | 651 | exons I- IV cDNA analysis | 1-2-0-0-3'-4-NA-NA |
| *SSH-A_974: femalePCR4 20_04_2018_974bp* | Female | 974 | exons I- IV cDNA analysis | 1-2-3-2'-3'-4-NA-NA |
| *SSH-W_969: femalePCR4 20_04_2018_969 bp* | Female | 969 | exons I- IV cDNA analysis | 1-2-3-2'-3'-4-NA-NA |
| *SSM-4_648: malePCR4 20_04_2018_648 bp* | Male | 648 | exons I- IV cDNA analysis | 1-0-0-2'-3'-4-NA-NA |
| *SSM-4_651: malePCR4 20_04_2018_651 bp* | Male | 651 | exons I- IV cDNA analysis | 1-0-0-2'-3'-4-NA-NA |
| *SSM-B_157: malePCR4 20_04_2018_157 bp* | Male | 157 | exons I- IV cDNA analysis | 1partial-0-0-0-0-4partial-NA-NA |
| *SSM-B_844: malePCR4 20_04_2018_844 bp* | Male | 844 | exons I- IV cDNA analysis | 1-2-3-2'-3'-4partial-NA-NA |
| *SSM-B_973: malePCR4 20_04_2018_973bp* | Male | 973 | exons I- IV cDNA analysis | 1-2-3-2'-3'-4-NA-NA |
| *SSM-W_651: malePCR4 20_04_2018_651 bp* | Male | 651 | exons I- IV cDNA analysis | 1-0-0-2'-3'-4-NA-NA |
| *SSM-W_972: malePCR4 20_04_2018_972 bp* | Male | 972 | exons I- IV cDNA analysis | 1-2-3-2'-3'-4-NA-NA |
| *SSM_16_163: malePCR4 20_04_2018_163pb* | Male | 163 | exons I- IV cDNA analysis | 1-0-0-0-0-4-NA-NA |
