# Supplementary material for: The genomic structure of the highly-conserved dmrt1 gene in Solea senegalensis (Kaup, 1868) shows an unexpected intragenic duplication
Source: PLoS One. 2020 Nov 2;15(11):e0241518. doi: 10.1371/journal.pone.0241518 (PMC7605655; doi:10.1371/journal.pone.0241518)
Supplement: S3 Table — A total of 143 transcripts were obtained from 45 fish species ranging from 1 to 10 in the species. (DOCX) [file pone.0241518.s004.docx]

**S3 Table:** **Number of *dmrt1* transcripts per 45 fish species obtained after data mining of Ensemble database.** A total of 143 transcripts were obtained from 45 fish species ranging from 1 to 10 in the species**.**

| Species | Transcripts |
| --- | --- |
| Amazon molly (*Poecilia formosa*) | 1 |
| Asian bonytongue (*Scleropages formosus*) | 1 |
| Bicolor damselfish (*Stegastes partitus*) | 1 |
| Channel catfish (*Ictalurus punctatus*) | 1 |
| Climbing perch (*Anabas testudineus*) | 1 |
| Cod (*Gadus morhua*) | 1 |
| Eastern happy (*Astatotilapia calliptera*) | 1 |
| Fugu (*Takifugu rubripes*) | 1 |
| Greater amberjack (*Seriola dumerili*) | 1 |
| Indian medaka (*Oryzias melastigma*) | 1 |
| Mangrove rivulus (*Kryptolebias marmoratus*) | 1 |
| Mexican tetra (*Astyanax mexicanus*) | 1 |
| Midas cichlid (*Amphilophus citrinellus*) | 1 |
| Paramormyrops kingsleyae (*Paramormyrops kingsleyae*) | 1 |
| Platyfish (*Xiphophorus maculatus*) | 1 |
| Red-bellied piranha (*Pygocentrus nattereri*) | 1 |
| Sailfin molly (*Poecilia latipinna*) | 1 |
| Sheepshead minnow (*Cyprinodon variegatus*) | 1 |
| Spiny chromis (*Acanthochromis polyacanthus*) | 1 |
| Spotted gar (*Lepisosteus oculatus*) | 1 |
| Tiger tail seahorse (*Hippocampus comes*) | 1 |
| Tilapia (*Oreochromis niloticus*) | 1 |
| Western mosquitofish (*Gambusia affinis*) | 1 |
| Zig-zag eel (*Mastacembelus armatus*) | 1 |
| Clown anemonefish (*Amphiprion ocellaris*) | 2 |
| Shortfin molly (*Poecilia mexicana*) | 2 |
| Stickleback (*Gasterosteus aculeatus*) | 2 |
| Tetraodon (*Tetraodon nigroviridis*) | 2 |
| Zebra mbuna (*Maylandia zebra*) | 2 |
| Swamp eel (*Monopterus albus*) | 3 |
| Tongue sole (*Cynoglossus semilaevis*) | 3 |
| Turbot (*Scophthalmus maximus*) | 3 |
| Japanese medaka HdrR (*Oryzias latipes*) | 4 |
| Japanese medaka HNI (*Oryzias latipes*) | 4 |
| Mummichog (*Fundulus heteroclitus*) | 5 |
| Ballan wrasse (*Labrus bergylta*) | 6 |
| Makobe Island cichlid (*Pundamilia nyererei*) | 6 |
| Burton's mouthbrooder (*Haplochromis burtoni*) | 7 |
| Northern pike (*Esox lucius*) | 9 |
| Zebrafish (*Danio rerio*) | 9 |
| Guppy (*Poecilia reticulata*) | 10 |
| Lyretail cichlid (*Neolamprologus brichardi*) | 10 |
| Orange clownfish (*Amphiprion percula*) | 10 |
| Periophthalmus magnuspinnatus (*Periophthalmus magnuspinnatus*) | 10 |
| Yellowtail amberjack (*Seriola lalandi dorsalis*) | 10 |
