# Supplementary material for: The genomic structure of the highly-conserved dmrt1 gene in Solea senegalensis (Kaup, 1868) shows an unexpected intragenic duplication
Source: PLoS One. 2020 Nov 2;15(11):e0241518. doi: 10.1371/journal.pone.0241518 (PMC7605655; doi:10.1371/journal.pone.0241518)

**S1 Fig:** **electrophoresis gel showing two amplification products after PCR using primers located in the cDNA duplicated region of the *S. senegalensis* *dmrt1* gene**. Lane 1: Hypper ladder 2 molecular weight marker (EcogenTM); Lane 2 and 3 PCR amplicons after using *Sse-F1* and *Sse-R1* primers.


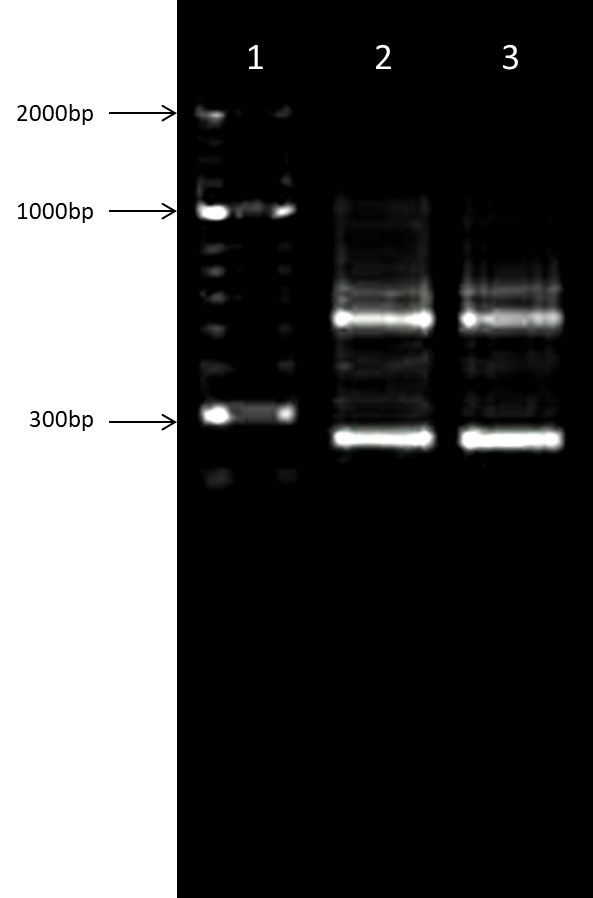

Supplement: S1 Fig — Lane 1: Hypperladder 2 molecular weight marker (EcogenTM); Lane 2 and 3 PCR amplicons after using Sse-F1 and Sse-R1 primers. (DOCX) [file pone.0241518.s005.docx]
