# Supplementary material for: The genomic structure of the highly-conserved dmrt1 gene in Solea senegalensis (Kaup, 1868) shows an unexpected intragenic duplication
Source: PLoS One. 2020 Nov 2;15(11):e0241518. doi: 10.1371/journal.pone.0241518 (PMC7605655; doi:10.1371/journal.pone.0241518)
Supplement: S2 Fig — Forty eight cDNA sequences of dmrt1 from males and females of S. senegalensis are shown. (DOCX) [file pone.0241518.s006.docx]

**
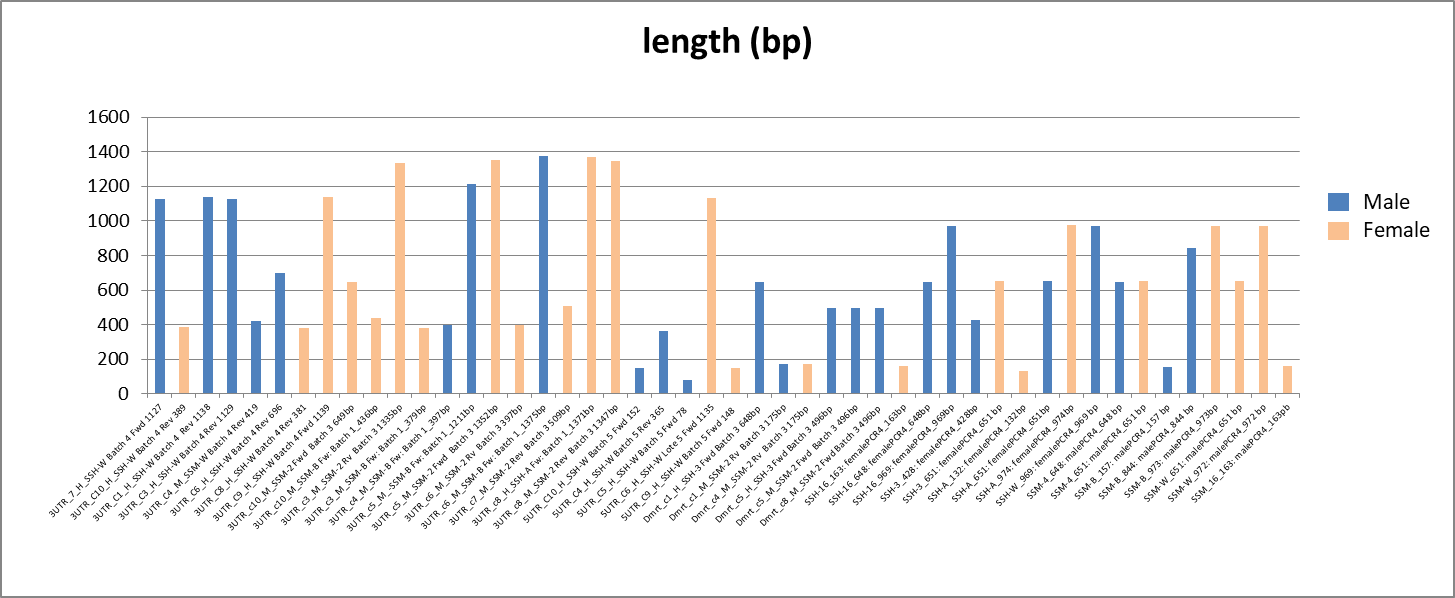
S2 Fig:** **Length of dmrt1 clone sequences in *S. senegalensis*.** Forty eight cDNA sequences of *dmrt1* from males and females of *S. senegalensis* are shown.
