# Supplementary material for: The genomic structure of the highly-conserved dmrt1 gene in Solea senegalensis (Kaup, 1868) shows an unexpected intragenic duplication
Source: PLoS One. 2020 Nov 2;15(11):e0241518. doi: 10.1371/journal.pone.0241518 (PMC7605655; doi:10.1371/journal.pone.0241518)

**S3 Fig:** **Alternative splicing in the *dmrt1* gene of S*olea senegalensis*.** Several different transcripts have been observed when using *primers* located in exons I, II, III (and therefore II’ and III’ as coming from the duplication event), and exon IV.


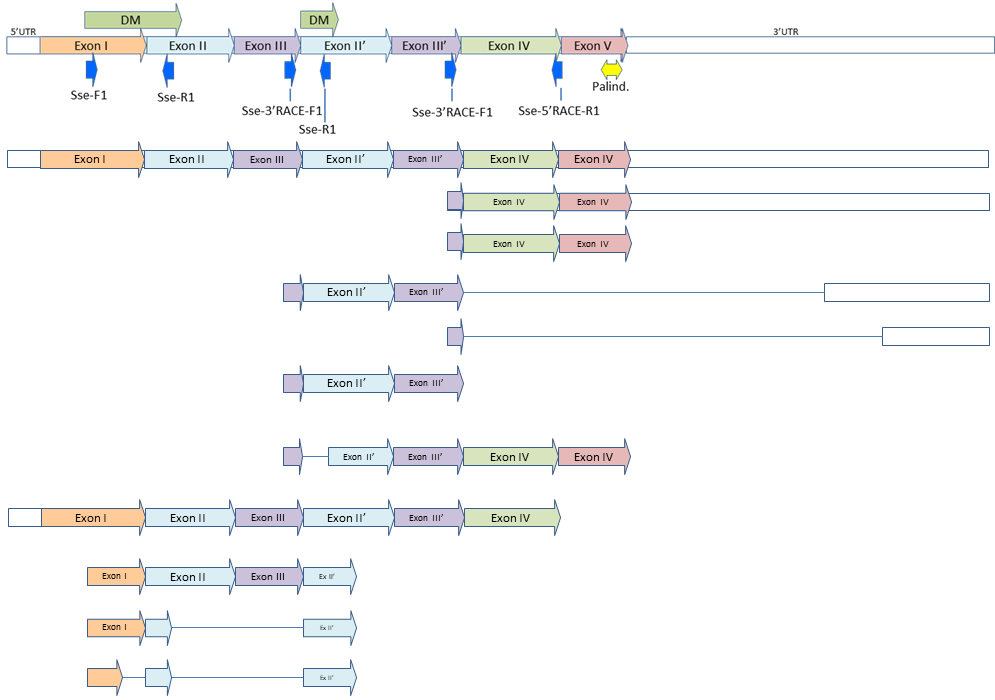

Supplement: S3 Fig — Several different transcripts have been observed when using primers located in exons I, II, III (and therefore II’ and III’ as coming from the duplication event), and exon IV. (DOCX) [file pone.0241518.s007.docx]
