# Supplementary material for: The genomic structure of the highly-conserved dmrt1 gene in Solea senegalensis (Kaup, 1868) shows an unexpected intragenic duplication
Source: PLoS One. 2020 Nov 2;15(11):e0241518. doi: 10.1371/journal.pone.0241518 (PMC7605655; doi:10.1371/journal.pone.0241518)
Supplement: S4 Fig — Several transcripts, with alternative exon splicing, are described from every species in Ensemble data base. (A) The gene sizes (kb) considering the sequence length comprising from 5’UTR to 3’UTR are displayed. (B) Basic statistics such as mean, median, mode, std. deviation, variance, range, minimum, maximum, percentiles are shown. (C) Box and whisker plot are displayed. Fish species: Amazon molly (Poecilia formosa), Asian bonytongue (Scleropages formosus), Bicolor damselfish (Stegastes partitus), Channel catfish (Ictalurus punctatus), Climbing perch (Anabas testudineus), Cod (Gadus morhua), Eastern happy (Astatotilapia calliptera), Fugu (Takifugu rubripes), Greater amberjack (Seriola dumerili), Indian medaka (Oryzias melastigma), Mangrove rivulus (Kryptolebias marmoratus), Mexican tetra (Astyanax mexicanus), Midas cichlid (Amphilophus citrinellus), Paramormyrops kingsleyae (Paramormyrops kingsleyae), Platyfish (Xiphophorus maculatus), Red-bellied piranha (Pygocentrus nattereri), Sailfin molly (Poecilia latipinna), Sheepshead minnow (Cyprinodon variegatus), Spiny chromis (Acanthochromis polyacanthus), Spotted gar (Lepisosteus oculatus), Tiger tail seahorse (Hippocampus comes), Tilapia (Oreochromis niloticus), Western mosquitofish (Gambusia affinis), Zig-zag eel (Mastacembelus armatus), Clown anemonefish (Amphiprion ocellaris), Shortfin molly (Poecilia mexicana), Stickleback (Gasterosteus aculeatus), Tetraodon (Tetraodon nigroviridis), Zebra mbuna (Maylandia zebra), Swamp eel (Monopterus albus), Tongue sole (Cynoglossus semilaevis), Turbot (Scophthalmus maximus), Japanese medaka HdrR (Oryzias latipes), Japanese medaka HNI (Oryzias latipes), Mummichog (Fundulus heteroclitus), Ballan wrasse (Labrus bergylta), Makobe Island cichlid (Pundamilia nyererei), Burton's mouthbrooder (Haplochromis burtoni), Northern pike (Esox lucius), Zebrafish (Danio rerio), Guppy (Poecilia reticulata), Lyretail cichlid (Neolamprologus brichardi), Orange clownfish (Amphiprion percula), Periop [file pone.0241518.s008.docx]

**S4 Fig:** **Size of *dmrt1* gene in 45 fish species.** Several transcripts, with alternative exon splicing, are described from every species in Ensemble data base. (A) The gene sizes (kb) considering the sequence length comprising from 5’UTR to 3’UTR are displayed. (B) Basic statistics such as mean, median, mode, std. deviation, variance, range, minimum, maximum, percentiles are shown. (C) Box and whisker plot are displayed. Fish species: Amazon molly (*Poecilia formosa*), Asian bonytongue (*Scleropages formosus*), Bicolor damselfish (*Stegastes partitus*), Channel catfish (*Ictalurus punctatus*), Climbing perch (*Anabas testudineus*), Cod (*Gadus morhua*), Eastern happy (*Astatotilapia calliptera*), Fugu (*Takifugu rubripes*), Greater amberjack (*Seriola dumerili*), Indian medaka (*Oryzias melastigma*), Mangrove rivulus (*Kryptolebias marmoratus*), Mexican tetra (*Astyanax mexicanus*), Midas cichlid (*Amphilophus citrinellus*), Paramormyrops kingsleyae (*Paramormyrops kingsleyae*), Platyfish (*Xiphophorus maculatus*), Red-bellied piranha (*Pygocentrus nattereri*), Sailfin molly (*Poecilia latipinna*), Sheepshead minnow (*Cyprinodon variegatus*), Spiny chromis (*Acanthochromis polyacanthus*), Spotted gar (*Lepisosteus oculatus*), Tiger tail seahorse (*Hippocampus comes*), Tilapia (*Oreochromis niloticus*), Western mosquitofish (*Gambusia affinis*), Zig-zag eel (*Mastacembelus armatus*), Clown anemonefish (*Amphiprion ocellaris*), Shortfin molly (*Poecilia mexicana*), Stickleback (*Gasterosteus aculeatus*), Tetraodon (*Tetraodon nigroviridis*), Zebra mbuna (*Maylandia zebra*), Swamp eel (*Monopterus albus*), Tongue sole (*Cynoglossus semilaevis*), Turbot (*Scophthalmus maximus*), Japanese medaka HdrR (*Oryzias latipes*), Japanese medaka HNI (*Oryzias latipes*), Mummichog (*Fundulus heteroclitus*), Ballan wrasse (*Labrus bergylta*), Makobe Island cichlid (*Pundamilia nyererei*), Burton's mouthbrooder (*Haplochromis burtoni*), Northern pike (*Esox lucius*), Zebrafish (*Danio rerio*), Guppy (*Poecilia reticulata*), Lyretail cichlid (*Neolamprologus brichardi*), Orange clownfish (*Amphiprion percula*), Periophthalmus magnuspinnatus (*Periophthalmus magnuspinnatus*), Yellowtail amberjack (*Seriola lalandi dorsalis*).


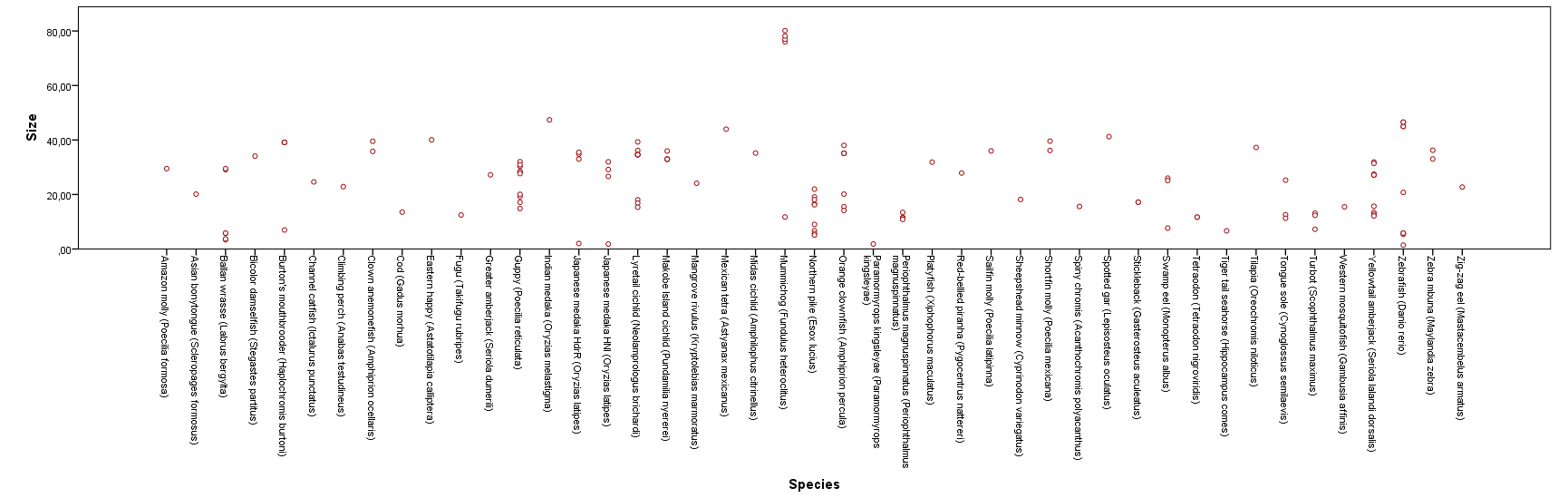
(A)

(B)

| N | | 143 | |
| --- | --- | --- | --- |
| Mean | 25,2813 | | |
| Median | 26,6200 | | |
| Mode | 11,42 | | |
| Std. Deviation | 15,04771 | | |
| Variance | 226,433 | | |
| Range | 78,71 | | |
| Minimum | 1,42 | | |
| Maximum | 80,13 | | |
| Percentiles | | 25 | 12,5800 |
|  |  | 50 | 26,6200 |
|  |  | 75 | 35,1100 |


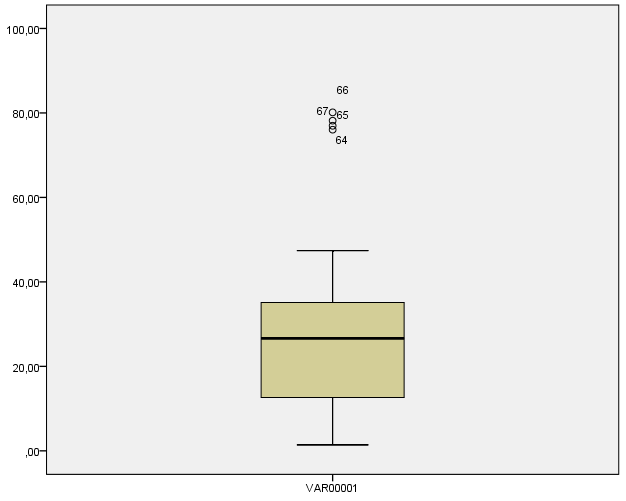
(C)
