# Supplementary material for: The genomic structure of the highly-conserved dmrt1 gene in Solea senegalensis (Kaup, 1868) shows an unexpected intragenic duplication
Source: PLoS One. 2020 Nov 2;15(11):e0241518. doi: 10.1371/journal.pone.0241518 (PMC7605655; doi:10.1371/journal.pone.0241518)

**S6 Fig:** **Duplicated intron alignments and plots of entropy as a measure of probability of positional homology in *dmrt1* gene of *S. senegalensis***. (A) Introns 1 and 1’ alignment and their plot of entropy; (B) Introns 2 and 2’ alignment and their plot of entropy;( C) Intron 3 and its duplicated region in intron 3’ alignment and their plot of entropy.

(A)

10 20 30 40 50 60 70 80

....|....|....|....|....|....|....|....|....|....|....|....|....|....|....|....|

***Sse_dmrt1_intron1***  **GTAACCGTTATTTCACCTGTTAGCGCGCGGCTAATTCAAAATTCAAAATTCAAAATAAAGAAGCGGTGACGAAATGAAAC**

***Sse_dmrt1_intron1’*** **GTAACCGTTATTTCACCTGTTAGCGCGCGGCT-------AATTCAAAATTCAAAATAAAGAAGCGGTGACGAAATGAAAC**

90 100 110 120 130 140 150 160

....|....|....|....|....|....|....|....|....|....|....|....|....|....|....|....|

***Sse_dmrt1_intron1***  **TCGAAACACAAAATGTAGCAGTTTGGATTAAATACCGTCGGTTCATGTTTACGCTGTTTAATAATAAGCTGACGTTCGTG**

***Sse_dmrt1_intron1’*** **TCGAAACACAAAATGTAGCAGTTTGGATTAAATACCGTTGGTTCATGTTTACGCTGTTTAATAATAAGCTCACATTCATT**

170 180 190 200 210 220 230 240

....|....|....|....|....|....|....|....|....|....|....|....|....|....|....|....|

***Sse_dmrt1_intron1***  **GTTTTCTCCTAACTTTCATTTGTGTACAAACAAGAGCTGCAGTTATGTATAGCTAGCTTGTTGGTAAACAACGTGTGTTT**

***Sse_dmrt1_intron1’*** **GTTTTCTCCTAACTTTCATTTGTGTACAAACAAGAGCTGCAGTTATGTATAGCTAGCTTGTTAGTAAACAACGTGTGTTT**

250 260 270 280 290 300 310 320

....|....|....|....|....|....|....|....|....|....|....|....|....|....|....|....|

***Sse_dmrt1_intron1***  **AGGGATGTCTTTACTATTTATTGTCTTGATTGTTGCACTCAAATAAGAGTTTAATTACAAAAACAAAGAAGTTATGTTTA**

***Sse_dmrt1_intron1’*** **AGGGATGTCTTTACTATTTATTGTATTGATTGTTGCACTCAAATAAGAGTTTAATTACAAAAACAAAGAAGTTATATTTA**

330 340 350 360 370 380 390 400

....|....|....|....|....|....|....|....|....|....|....|....|....|....|....|....|

***Sse_dmrt1_intron1***  **ATTTATTGCTCTCTGATGTTGCAGTTTGTTGCTTGAGACTACTTTTTTGGCTTAAGAGAATAATGTTATGATCATTGCTA**

***Sse_dmrt1_intron1’*** **ATTTATTGCTCTCTGATGTTGCAGTTTGTTGCTTGAGGCTACTTTTTTGGCTTAAGAGAATAATGTTATGATCATTGCTA**

410 420 430 440 450 460 470 480

....|....|....|....|....|....|....|....|....|....|....|....|....|....|....|....|

***Sse_dmrt1_intron1***  **GTTTGAGGTGAGGTTACTTGAGATGAAAACTACATTATACAGTAGATGATATTTTTTGTGTATTTGTTGTATGACATGAG**

***Sse_dmrt1_intron1’*** **GTTTGAGGTGAGGTTACTTGAGATGAAAACTACATTATACAGTAGATGATCTTTTTTGTGTATTTGTTGTATGACATGAG**

490 500 510 520 530 540 550 560

....|....|....|....|....|....|....|....|....|....|....|....|....|....|....|....|

***Sse_dmrt1_intron1***  **TAAGTTATTATTGATTATCAAGACATTTGCTGACAGATCATCGTTTCCGTCTATGGTATTTGTGCTCATAAGTCT-----**

***Sse_dmrt1_intron1’*** **TAAGTTATTATTGATTATCAAGACATTTGCTGACAGATCATCGTTTCCGTCTATGGTATTTGTGTTCATAAGTCTGCAGT**

570 580 590 600 610 620 630 640

....|....|....|....|....|....|....|....|....|....|....|....|....|....|....|....|

***Sse_dmrt1_intron1***  **--------------------------------------------------------------------------------**

***Sse_dmrt1_intron1’*** **GTATATTGACATGACATTGTTGATTCATCTTTACACAGTTCGTGTAATGACTTTCTAATGAGTCTGGTTATTATTGTGTA**

650 660 670 680 690

....|....|....|....|....|....|....|....|....|....|....|

***Sse_dmrt1_intron1***  **--------------------------TATCAGTGTTCTCCTTTCCTCCCATCCAG**

***Sse_dmrt1_intron1’*** **GCAGGTAGTGAGTCTGCCCTGTGTCTTATCAGTGTTCTCCTTTCCTCCCATCCAG**


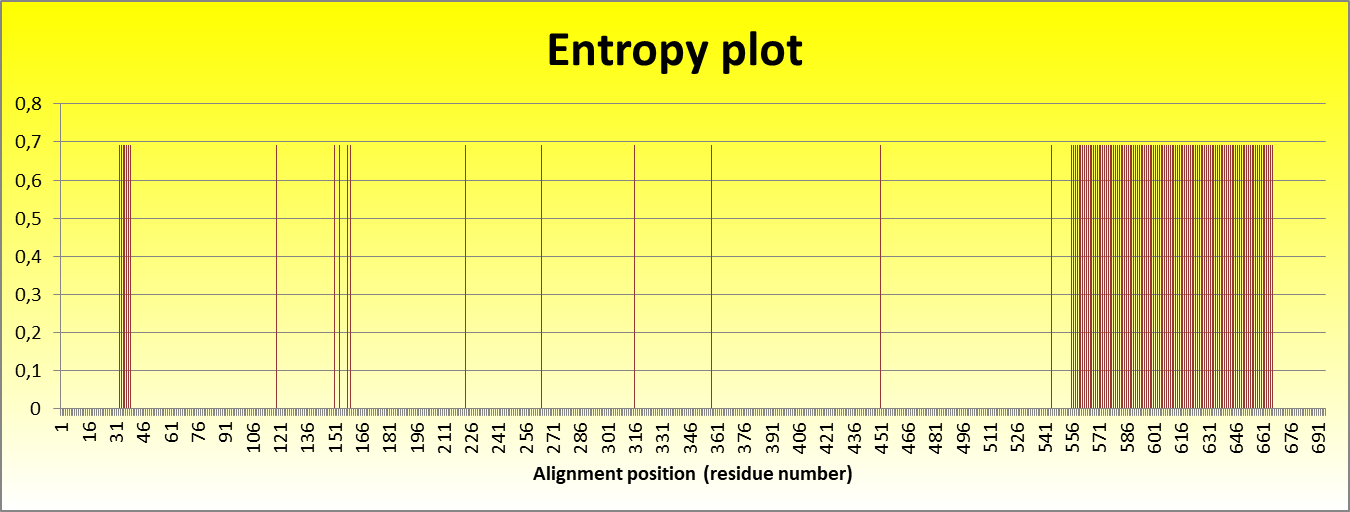


(B)

10 20 30 40 50 60 70 80

....|....|....|....|....|....|....|....|....|....|....|....|....|....|....|....|

***Sse_dmrt1_intron2***  **GGTGAGGCATTTGCAAGTGCTCTTTTACCTGCTATCACACCACACCCCTTATATTATGTGTCTCACCACCAGTCAACAGC**

***Sse_dmrt1_intron2’*** **GGTGAGGCATTTGCAAGTGCTCTTTTACCTGCTATCACACCACACCCCTTATATTATGTGTCTCACCACCAGTCAACAGC**

90 100 110 120 130 140 150 160

....|....|....|....|....|....|....|....|....|....|....|....|....|....|....|....|

***Sse_dmrt1_intron2***  **AGCACTCTCTACAAACTAAACCTTTTTCCTGAACCATAAATTCACCAGAGCCACCAAATGTTAAGGCAGAGAGTGTCTTG**

***Sse_dmrt1_intron2’*** **AGCACTCTCTACAAACTAAACCTTTTTCCTGAACCATAAATTCACCAGAGCCACCAAATGTTAAGGCAGAGAGTGTCTTG**

170 180 190 200 210 220 230 240

....|....|....|....|....|....|....|....|....|....|....|....|....|....|....|....|

***Sse_dmrt1_intron2***  **ACCTCCCTGGGCCTGATTCTGGCAGTTTTATTCTCTGACTATTATAAAGTCAGATGCCATTCAGACTTTATATTAACATC**

***Sse_dmrt1_intron2’*** **ACCTCCCTGGGCCTGATTCTGGCAGTTTTATTCTCTGACTATTATAAAGTCAGATGCCATTCAGACTTTATATTAACATC**

250 260 270 280 290 300 310 320

....|....|....|....|....|....|....|....|....|....|....|....|....|....|....|....|

***Sse_dmrt1_intron2***  **TGTCCTCCATTATCATGTCTAGACTGTGTTAAAATGCAATCACACAAACCGTATTTGGAGCTGATTTGAGTGGCCACATT**

***Sse_dmrt1_intron2’*** **TGTCCTCAGTTATCATGTCTAGACTGTGTTAAAATGCAATCACACAAACCGTATTTGGAGCTGATTTGAGTGGCCACATT**

330 340 350 360 370 380 390 400

....|....|....|....|....|....|....|....|....|....|....|....|....|....|....|....|

***Sse_dmrt1_intron2***  **TACTGAGCTGCATGAATGCAATCCATCCTCAGGACACAGTAGTAGTAATACCTCAGCTACCACCACAGTATTAACACTGA**

***Sse_dmrt1_intron2’*** **TACTGAGCTGCATGAATGCAATCCATCCTCAGGACACAGTAGTAGTAATACCTCAGCCACCACCACAGTATTAACACTGA**

410 420 430 440 450 460 470 480

....|....|....|....|....|....|....|....|....|....|....|....|....|....|....|....|

***Sse_dmrt1_intron2***  **TTACTAGGGCTGCATGATACATCTTAAATAAAGATCTTGATCTCGATTCACAACTACACGTGATCTCATTTCTAAATGAC**

***Sse_dmrt1_intron2’*** **TTACTAGGGCTGCATGATACATCATAAATAAA---------------------------CTAATGCAACGATGGAAGGAC**

490 500 510 520 530 540 550 560

....|....|....|....|....|....|....|....|....|....|....|....|....|....|....|....|

***Sse_dmrt1_intron2***  **AACGATTCTTCTGCATTTTATTTCATGAGCTCATCGCAAATAAACAATCCTATTTTGCTCTATATGAAGCGCCATTACAT**

***Sse_dmrt1_intron2’*** **AGAGGTTCAGT---------------------------------------------------------------------**

570 580 590 600 610 620 630 640

....|....|....|....|....|....|....|....|....|....|....|....|....|....|....|....|

***Sse_dmrt1_intron2***  **CACGTGATTCAGTGGAGACATTTCCTTTGCTGGAGATGATCCCACGTTTTCAGGATTGTTAAGTCTTTTTAACTGATCTA**

***Sse_dmrt1_intron2’*** **-----------GTGCAACAAGGTTCCATTCTCAAGTTAGTTGCCCATCAAAATGATTCTGAGGCTTT-----------GA**

650 660 670 680 690 700 710 720

....|....|....|....|....|....|....|....|....|....|....|....|....|....|....|....|

***Sse_dmrt1_intron2***  **CAGTTCGGCATTGTTCAGTTTGATTCTTGTGCCTCTTCCCATGTCGGCACTCTGACCAATCAGTGCACGGCAGTCTGACG**

***Sse_dmrt1_intron2’*** **TTTTTTGGGGTAGAAAGGTTTGCATCGTGTTGTTCAAAACTTGATTACACCAAAAAAA----------------------**

730 740 750 760 770 780 790 800

....|....|....|....|....|....|....|....|....|....|....|....|....|....|....|....|

***Sse_dmrt1_intron2***  **ACGTCTCGCGTGTCCCCTGGTTATGACATCCCAAGTCGAGCTAGTGGAAACACGCCATATGTTACTTAAGCCACTACAAT**

***Sse_dmrt1_intron2’*** **--------CGTGTCTTAAAGTTGCAGTAGACAG-----------------------GTACTTCAGAAAAATCATTAAATC**

810 820 830 840 850 860 870 880

....|....|....|....|....|....|....|....|....|....|....|....|....|....|....|....|

***Sse_dmrt1_intron2***  **AATAACAACAACAATAACAATTATAACAATAAGCCCATTTTGCAGGGACATGTTCAGGGCCTAAATCACACTGTTTGGCA**

***Sse_dmrt1_intron2’*** **AGGAGAGATAA-----------GTGAAAGTAAATTAACTAAACAGGGGGAGATATGG-----------------TGGTGA**

890 900 910 920 930 940 950 960

....|....|....|....|....|....|....|....|....|....|....|....|....|....|....|....|

***Sse_dmrt1_intron2***  **CTTTAATTGAATGCCTAATTGAACACACACACACACAAACAAGCTGACAGCGCAATTTTTTTGTGGACTGTATTTGAATA**

***Sse_dmrt1_intron2’*** **TGGTGGTTGGTAACCTGATGGGACACAGATGGAGGATGAGGGATGGTGGATCCAATGACACTGTGAAGACTCAGGAAGTG**

970 980 990 1000 1010 1020 1030 1040

....|....|....|....|....|....|....|....|....|....|....|....|....|....|....|....|

***Sse_dmrt1_intron2***  **AAGGTTGGGAACACTAGTTCTCATTTTATTTTAAAAATAATAAAATTTAATAATACATAATAATATTTTATTTTAATATA**

***Sse_dmrt1_intron2’*** **GCAGCTGTGG----------------------AGTGACAGTGCATGTGCACAAGAGGAGATGAAAGCC--------TGGA**

1050 1060 1070 1080 1090 1100 1110 1120

....|....|....|....|....|....|....|....|....|....|....|....|....|....|....|....|

***Sse_dmrt1_intron2***  **TAATTTTAATAAAGGTTTTATTTAACCTTTATTTAATAATGTTCCTACCTGCTCATCGAACGTACATGATGCAAGGATGG**

***Sse_dmrt1_intron2’*** **GAATGTGAATAAGGCT----------GATCGATTACAACAGTGTCGTCGTCCTGACTGAACTTACTGTAGAGCTGAATTT**

1130 1140 1150 1160 1170 1180 1190 1200

....|....|....|....|....|....|....|....|....|....|....|....|....|....|....|....|

***Sse_dmrt1_intron2* AAGGACAGAGTTTCAGAGTGCAACATGGTTCCATTCTCAAGTTAGTTACCCATCGAAATGATTGTGAGGCTGTGATTTTT**

***Sse_dmrt1_intron2’* ATTTGTTGAGTCTCACACAGATACATGGC---------------ATGTCATGTGTGACCTGTGGCGACCTCTCTATCCCT**

1210 1220 1230 1240 1250 1260 1270 1280

....|....|....|....|....|....|....|....|....|....|....|....|....|....|....|....|

***Sse_dmrt1_intron2* GGGGAAGAAAGGTTTGCATAATTTTTTTGACAGGTTTATTGTCCGCCATGTTTTCTCTTTATTTTCAGTTTATACAGTTG**

***Sse_dmrt1_intron2’* GAAG---------CTCACCACTCATTTTGACAGGTTTATAGTCCGCCATGTTTTCTCTTTATTTTC--TTTACTTAGTTG**

1290 1300 1310 1320 1330 1340 1350 1360

....|....|....|....|....|....|....|....|....|....|....|....|....|....|....|....|

***Sse_dmrt1_intron2* TTGCAGTTTAATAGTATCACTTTCACCAAATGAACATGTATATACAACTCCTGTATATCATGTATATCCAACAGCCAAAA**

***Sse_dmrt1_intron2’* TTGCAATTTAATAGTATCACTTTCACCAAATGAACATGTATATCCAACTCCTTAGTA-----------GAACAGCCAAAA**

1370 1380 1390 1400 1410 1420 1430 1440

....|....|....|....|....|....|....|....|....|....|....|....|....|....|....|....|

***Sse_dmrt1_intron2* AGAGCACCCTCCTTATTTGAAATCCCCCTGTCATGGGCCAGATGTCCCTCAGACTGTGAAAAGAGCCTGTAGTCTAGTTT**

***Sse_dmrt1_intron2’* AGAGCACCCTCCTTATTTGAAATCCCCCTGTCATGGGCCAGATGTCCCTTAGACTGTGAACAGAGCCTGTATTCTAGTTT**

1450 1460 1470 1480 1490 1500 1510 1520

....|....|....|....|....|....|....|....|....|....|....|....|....|....|....|....|

***Sse_dmrt1_intron2* AGTTTT--------------------------------------------------------------------------**

***Sse_dmrt1_intron2’* AGTTTTCATTGAGATCACCTGATCTACATTATGCTGAAAAGTTATCATAGAATTATTGATACAAAAAATGCCAAAAATAT**

1530 1540 1550 1560 1570 1580 1590 1600

....|....|....|....|....|....|....|....|....|....|....|....|....|....|....|....|

***Sse_dmrt1_intron2* ------------------TAAGAGAAGCGTTTTCTCTGTTAGTTTTCTGTACATTGTTATGGAATCCTCTTATTTGATTT**

***Sse_dmrt1_intron2’* AATTTTTTTCACCCAGTGTAAGAGACGCGTTTTCTCTGTTAGTTTTCTGTACATTGTTATGGAATCCTCTTATTTGATTT**

1610 1620 1630 1640 1650 1660 1670 1680

....|....|....|....|....|....|....|....|....|....|....|....|....|....|....|....|

***Sse_dmrt1_intron2* TTCATATGGAAGACAGGACCATAGAAAAGACTCTCTTTTCTTCTCATGTGGAGGCAGACCCTGATAGCTGTGTTCCCACT**

***Sse_dmrt1_intron2’* TTCATATGGAGGACAGGAACATAGAAAAGACTCTCTTTTCTTCTCATGTGGAGGCAGACCCTGATAACTGTGTTCCCACT**

1690 1700 1710 1720 1730 1740 1750 1760

....|....|....|....|....|....|....|....|....|....|....|....|....|....|....|....|

***Sse_dmrt1_intron2* CCCACTCAGTCTGATAGCAACATTACAGGAGGTGGAATACAGCGTGGTATTTGTGGTGAGCTTAGGGTCCCTGACATGGC**

***Sse_dmrt1_intron2’* CCCACTCAGTCTGATAGCAACATTACAGGAGGTGGAATACAGCGTGGTATTTGTGGTGAGCTTAGGGTCCCTGACATGGC**

1770 1780 1790 1800 1810 1820 1830 1840

....|....|....|....|....|....|....|....|....|....|....|....|....|....|....|....|

***Sse_dmrt1_intron2* AACAGAGAGAACAAAGTGAGGTGGAAAAAGGAGTTCAGCACTGTGTGATGCACTGATGACAAGAGAACAGGGACAGCATA**

***Sse_dmrt1_intron2’* AACAGAGAGAACAAAGTGAGGTGGAAAAAGGAGTTCAGCACTGTGTGATGCACTGATGACAAGAGAACAGGGACAGCATA**

1850 1860 1870 1880 1890 1900 1910 1920

....|....|....|....|....|....|....|....|....|....|....|....|....|....|....|....|

***Sse_dmrt1_intron2* CGCATGACATTATCTTTGGCTGCACTTGTGCACAGTGTCTTCATTGTGCGAAGGCAAAAGAGCAGGGTGTTCCTGACGAG**

***Sse_dmrt1_intron2’* CGCATGACATTATCTTTGGCTGCACTTGTGCACAGTGTCTTCATTGTGCGAAGGCAAAAGAGCAGGGTGTTCCTGACGAG**

1930 1940 1950 1960 1970 1980 1990 2000

....|....|....|....|....|....|....|....|....|....|....|....|....|....|....|....|

***Sse_dmrt1_intron2* AATGTGGCCTTGTGGATAAAGAAAATACAAGAGATAATAAGCAATGACGATTTAATGGTCTCATATGTGCTGACAGATTC**

***Sse_dmrt1_intron2’* AATGTGGCCTTGTGGATAAAGAAAATACAAGAGATAATAAGCAATGACGATTTAATGGTCTCATATGTGCTGATAGATTC**

2010 2020 2030 2040 2050 2060 2070 2080

....|....|....|....|....|....|....|....|....|....|....|....|....|....|....|....|

***Sse_dmrt1_intron2* ATACCTGTAAGGTGGTGGCACGGTGTGGGAGGTCGGCAGGAAGTTGCAGTTGTCACCTGCAGATGGTGCTGTTGTCAGTC**

***Sse_dmrt1_intron2’* ATACCTGTAAGGTGGTGGCACGGTGTGGGAGGTCGGCAGGAAGTTGCAGTTGTCACCTGCAGATGGTGCTGTTGTCAGTC**

2090 2100 2110 2120 2130 2140 2150 2160

....|....|....|....|....|....|....|....|....|....|....|....|....|....|....|....|

***Sse_dmrt1_intron2* ATTTGAGCCAAGGCTGGTGTTACAGA-------AAGTTCAATTACTGTAATGTATTTTTTTTATATAAG-----------**

***Sse_dmrt1_intron2’* ATTTGAGCCAAGGGTGGTGTTACAGAGAAAGTGAAGTTCAATTACTGTAATGTTTTTTTTATTAATAGAGCTAGTAAATA**

2170 2180 2190 2200 2210 2220 2230 2240

....|....|....|....|....|....|....|....|....|....|....|....|....|....|....|....|

***Sse_dmrt1_intron2* --------TTATATAATTGTTCATGTCAGCTTTTGTGTTGTCTTGGTTGTTTATTAGGTCTTCTGCTAGATGCATGGAAT**

***Sse_dmrt1_intron2’* TTTTTAACTTATATAATTGTTCATGTCAGCTTTTGTGTTGTCTTGGTTGTTTATTAGGTCTGCTGCTAGATGCATGGAAT**

2250 2260 2270 2280 2290 2300 2310 2320

....|....|....|....|....|....|....|....|....|....|....|....|....|....|....|....|

***Sse_dmrt1_intron2* TGAAAACACACATACC-TTTTTTATATAAGTTATATAATTGTTGATGTCACCTTTTGTGTTGTCATCGTTTAAGTGTCCC**

***Sse_dmrt1_intron2’* TGAAAACACACATACCTTTTTTTATATAAGTTATATAATTGTTGATGTCACCTTTTGTGTTGTCATTGTTTAAGTGTCCC**

2330 2340 2350 2360 2370 2380 2390 2400

....|....|....|....|....|....|....|....|....|....|....|....|....|....|....|....|

***Sse_dmrt1_intron2* ATGCACTCACAGACAACATCATTCATCCTTTGACTGGAATGTTGTCTTATTTGCTGTCTAATCTCTGTATGTTTTGGTTC**

***Sse_dmrt1_intron2’* ACTCACTCACAGACAACATCATTCATCCTTTAAATGGAATGTTGTCTTATTTGCTGTCTAATCTCTGTATGTTTTGGTTC**

.

***Sse_dmrt1_intron2* A**

***Sse_dmrt1_intron2’* A**


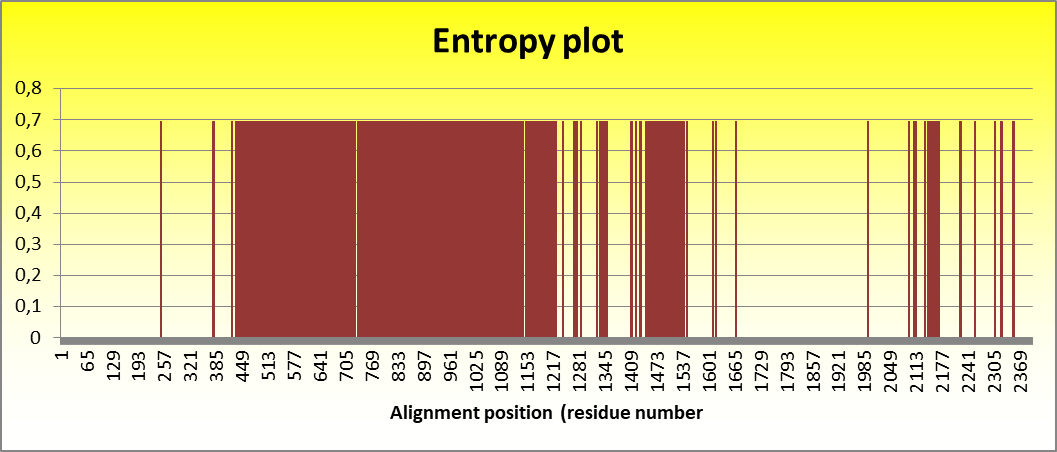


(C)

10 20 30 40 50 60 70 80

....|....|....|....|....|....|....|....|....|....|....|....|....|....|....|....|

***Sse_intron3***  **GTGACTAACATCATTCTCCATGCTTGATTATCTGTAACACTTGAGTATTTAATAGCCCTTTTTTAAACATCTGGTAGGTA**

***Sse_intron3’***  **GTGACTAACATCATTCACCATGCTTGATTATCTGTAACACTTGAGTATTTAATAGCCCTTTTTTAAACATCTGGTAGGTA**

90 100 110 120 130 140 150 160

....|....|....|....|....|....|....|....|....|....|....|....|....|....|....|....|

***Sse_intron3***  **TTCTATTGCAGTATACATGTTAACCCCCCCCCCATGCATATTAATATGAATAGGAAGCATTGTTGTTTAACTTGTGCTGC**

***Sse_intron3’***  **TTCTATTGCAGTACACACGTTAACCCCCCCC-------------------------------------------------**

170 180 190 200 210 220 230 240

....|....|....|....|....|....|....|....|....|....|....|....|....|....|....|....|

***Sse_intron3***  **TTAGGGACAGAAGGGATGTGTGTACTACATTACAAATTATATTCTTATTTATAGTCTCCTCTGTTCAAAATGACTGACAC**

***Sse_intron3’***  **-------------------------------------------------------------------------------C**

250 260 270 280 290 300 310 320

....|....|....|....|....|....|....|....|....|....|....|....|....|....|....|....|

***Sse_intron3***  **CAGGATTCTTCTGTTTTAGGGTTTTCAAGATTGTTATGACATTGATTTTTTTATTATTTATTTCAATTATTTAATGTAAT**

***Sse_intron3’***  **CAGGATTCTTCTATTTTAGGGTTTTCAAGATTGTTATGGCACTGATCATT----------------TTATTTAATGTAAT**

330 340 350 360 370 380 390 400

....|....|....|....|....|....|....|....|....|....|....|....|....|....|....|....|

***Sse_intron3***  **TCAAATGGACATTTTAATGACAAGATTCAAGTCAGCATAGCTTTATTGAACTGCTTTAATACCAAAATATGTTTGGGATT**

***Sse_intron3’***  **CCATATGGACATTTTAATGACAAGATTCAAGTCAGCATAGCTTTATTGAACTGCTTTTATACCAGAATGAGTTTGGGATT**

410 420 430 440 450 460 470 480

....|....|....|....|....|....|....|....|....|....|....|....|....|....|....|....|

***Sse_intron3***  **AATCACTACATG------CAAAAAACAAAACACAATTTCAAATTTCTGGCAACTTATTTTCC------------------**

***Sse_intron3’***  **AATCACTTCATGTAAAAATAAAAAACAAAACAGAATTTCAATTTCTTATTTTCCACTGTTTCCACTGTCAGAAAGAATTC**

490 500 510 520 530 540 550 560

....|....|....|....|....|....|....|....|....|....|....|....|....|....|....|....|

***Sse_intron3***  **------------------------ATATTTTTGGAAATTATTTAATAAATGGAAGATTTGAAACAATGGAAACAATGTAG**

***Sse_intron3’***  **ACCGATCATAAAAGAAAGAGTAGAATGTGCTTGGAAATGATTTGATAAATGGAAGATTT---------GAAACAATGTAG**

570 580 590 600 610 620 630 640

....|....|....|....|....|....|....|....|....|....|....|....|....|....|....|....|

***Sse_intron3***  **CTAAGAAATTGCAAAGGTTGCAGTTTACGACATATTCTGTATTATGCCGCACATCTGACCCTGGTGTTTTTATTAAACGT**

***Sse_intron3’***  **CTAACAAATTGCAAAGGTTGCAGTTTACGACATATTCTGTATTATGCCGCACATCTGACCCTGGTGTTTTTATTAAATGT**

650 660 670 680 690 700 710 720

....|....|....|....|....|....|....|....|....|....|....|....|....|....|....|....|

***Sse_intron3***  **CAATGAAAGGTCCTTTTATTTTTTTTTATAATTCATAAATGTCTGGAGCAAATTAAAATATTGTTGTTGTTTAATTGTTA**

***Sse_intron3’***  **CAATGAAAGGTCC----------TTTTATAATTCATAAATGTCCGGAGCAAATTAAAATATTGTTGTTGTTTAATTGTTA**

730 740 750 760 770 780 790 800

....|....|....|....|....|....|....|....|....|....|....|....|....|....|....|....|

***Sse_intron3***  **TTAAGTCTTATTAGGTTCCCATCCAGTACTGATGATGTAAGGCTGCATTACATTACAAACCTATAATACAGTGGATATTG**

***Sse_intron3’***  **TTAAGTCT---------------CAGTACTGATGATGTAAGGCAGCATTAGGTTACAAACCTATAATACAGTGGATATTG**

810 820 830 840 850 860 870 880

....|....|....|....|....|....|....|....|....|....|....|....|....|....|....|....|

***Sse_intron3***  **TACAAAAGCTTTTCTTTCTAATTATATAAATGATATCACACACAATCACAATTATTCATTCTACCCAAATCGTTCAACCC**

***Sse_intron3’***  **TACAAAAGCTTTTCTTTCTAATTATATAAATGATAT----CACAATCACAATTATTCATTCTACCCAAATCGTTCAACCC**

890 900 910 920 930 940 950 960

....|....|....|....|....|....|....|....|....|....|....|....|....|....|....|....|

***Sse_intron3* GATACCAGATACCACTTGAGTTGCAGTCAGCACTTACCTACATTAAAAATAGCACGGTAACGTTTGTGTAAGCTCATATG**

**Sse_intron3’**  **AATACAAGATACAACTTGAGTTGCAGTCAGCACTTACCTTCATTAAAAATAGCATGGTAACGTTTGTGTAAGCTCATATG**

970 980 990 1000 1010 1020 1030 1040

....|....|....|....|....|....|....|....|....|....|....|....|....|....|....|....|

***Sse_intron3* TTATGGCATAGAGTGATGGTACACTATTGTTGACTGCTCTTCAACTGAAAGATTTAGGATTCAGCTTGTGACCAGTCACA**

**Sse_intron3’**  **TTATGGCATAGAGTGATGGTACACTAGTGTTGACTGCTCTTCAACTGAAAGATTTAGGATTCAGCTTGTGACCAGTCACA**

1050 1060 1070 1080 1090 1100 1110 1120

....|....|....|....|....|....|....|....|....|....|....|....|....|....|....|....|

***Sse_intron3* GGAGTCATGTGCAAGAGTTAAAGAACAAAGGCAAAAGTCCTGCCGCTGTGCATTAGAGGAGATGGATTGGGCGTACAGTA**

***Sse_intron3’* GGAGTCATGTGCAAGAGTTAAAGAACCAAGGCAAAAATCCTGCAGCTGTGCATTAGAGGAGATGGATTGGGCGTACAGTA**

1130 1140 1150 1160 1170 1180 1190 1200

....|....|....|....|....|....|....|....|....|....|....|....|....|....|....|....|

***Sse_intron3* AGAAATGTAATGATGTTGTGTTTGTGTACGTGCGTT--------------------------------------------**

***Sse_intron3’* AGAAATGTAATGATGTTGTGTTTGTGTATGTGCGGTTTTCAGGCCATAGACAGACCAACAAGTCAGCGCAGCATGCAAAA**

1210 1220 1230 1240 1250 1260 1270 1280

....|....|....|....|....|....|....|....|....|....|....|....|....|....|....|....|

***Sse_intron3* ------------------------------------------------------------TGTGTGTGCGCAC-------**

***Sse_intron3’* GGTTCACCTGATCGTTATATCACCGTTACACTAATAAAAAAGACTCATATCAGTGTGAGCTGTGTGGATGCACAGTGGAA**

1290 1300 1310 1320 1330 1340 1350 1360

....|....|....|....|....|....|....|....|....|....|....|....|....|....|....|....|

***Sse_intron3* --------------------------------------------------------------------------------**

***Sse_intron3’* AGCGATGTACACATTATATCACACCAAAACCATTGGTGTCTAATTAGTAAATAAATAAGTTAAATAATAAAACCTTTTTT**

1370 1380 1390 1400 1410 1420 1430 1440

....|....|....|....|....|....|....|....|....|....|....|....|....|....|....|....|

***Sse_intron3* --------------------------------------------------------------------------------**

***Sse_intron3’* TTTAATTTAACTATGGCTCTCTGTATACCAAATGTTGAATTTCCTTCTCTATTTAAAAAAGATAAAAAAATAAATCACCC**

1450 1460 1470 1480 1490 1500 1510 1520

....|....|....|....|....|....|....|....|....|....|....|....|....|....|....|....|

***Sse_intron3* --------------------------------------------------TAAGCTGTTTTCATGGCACA----------**

***Sse_intron3’* GAGCCTGTCGTCACCCGACAGTTCGGTGACATGAATTGACATGTTAATTTCAAGTTCTTTTATTGAAACATAGAGTAACA**

1530 1540 1550 1560 1570 1580 1590 1600

....|....|....|....|....|....|....|....|....|....|....|....|....|....|....|....|

***Sse_intron3* --------------------------------------------------------------------------------**

***Sse_intron3’* CAGGGTTCAAATGGCTTTTTTAAATTGCCGTATTGAAAATATGGCGTAAAGCTAATGAAGCCACAATGGTTGGATGATGT**

1610 1620 1630 1640 1650 1660 1670 1680

....|....|....|....|....|....|....|....|....|....|....|....|....|....|....|....|

***Sse_intron3* ------------------------------------------------GACAGACCATACA-------------------**

***Sse_intron3’* AAAAAGTTATCAGCGGTTACAGTTAATCAAGAGATAAAGAGGGATAATAAGAGATCATAAAGAAGAACTGATTGTTGCTA**

1690 1700 1710 1720 1730 1740 1750 1760

....|....|....|....|....|....|....|....|....|....|....|....|....|....|....|....|

***Sse_intron3* --------------------------------------------------------------------------------**

***Sse_intron3’* ATGGTACGTCTGAAATTTTATGATACTCATAAGTGCTGGTGATAACTCATAAGATGTGTGTCTCTGTGGGTTCATGGGGG**

1770 1780 1790 1800 1810 1820 1830 1840

....|....|....|....|....|....|....|....|....|....|....|....|....|....|....|....|

***Sse_intron3* -----------TTCCTCCTGAATGTAACGATCATTTCTGTCATTTGCTGTAACCGTGAATAAATGTTAACTGTAAATGTT**

***Sse_intron3’* CTCATAGTTGCTTCCTCCTGAATGTAACGAGCATTTCTGTCATTTGCTGTAACCGCGAATAAATGTTAACAACCACCTAC**

1850 1860 1870 1880 1890 1900 1910 1920

....|....|....|....|....|....|....|....|....|....|....|....|....|....|....|....|

***Sse_intron3* AAA----------------TGTCTTACTGTAGCTTTATCAGGGGATTTTTTGCGCTCAGAAAAAAACAAAAGTAAGACGT**

***Sse_intron3’* ATGACAGTCCGAGTGCGCTTGTCTTACTGTAGCTGTATC-GGGTATTTTTTGCGCTTAGAAAAAAATAAAAATAGGACGT**

1930 1940 1950 1960 1970 1980 1990 2000

....|....|....|....|....|....|....|....|....|....|....|....|....|....|....|....|

***Sse_intron3* GTGTCAGATACTGTCAATACTCCCAT-GGTTCATGTCGGGTTCATGTTGGGTTTGGGCAGAAAAAAGCGGCCCAGGCCGT**

***Sse_intron3’* GTGTCAAATACTGTCAATACTCCCATAGGTTCATGTCGGGTTCATGTCGGGTTCGGGCAGAAAAAAGCGGCCCAGGCCGT**

2010 2020 2030 2040 2050 2060 2070 2080

....|....|....|....|....|....|....|....|....|....|....|....|....|....|....|....|

***Sse_intron3* GCTCTATTCCAGGCTTATGTTGCACAT-----------CTCGCCCTTTTTTCGCTGTCATTTGTCTGTGCTGTCAGCTTA**

***Sse_intron3’* GCTCTATTCCAGGCTTATGTTGCACATCACGCCCTTCACTCGCCCTTTTTTCGCTGTCATTTGTCTGTGCTGTCAGCTTA**

2090 2100 2110 2120 2130 2140 2150 2160

....|....|....|....|....|....|....|....|....|....|....|....|....|....|....|....|

***Sse_intron3* TGAATGAAG--------GCCCTAAAAGTAGACTTAAACACATTTAGACTTGCAAGTAAAAAAAACTGTGGAAAAATGTAA**

***Sse_intron3’* TGAATGAAGGCCAAAGTGCCCTAAAAGTAGACTTAAACACATTTAAA----CAAGTAAAAAAAACAGTGGAAAAAATTAA**

2170 2180 2190 2200 2210 2220 2230 2240

....|....|....|....|....|....|....|....|....|....|....|....|....|....|....|....|

***Sse_intron3* AATTAGCAACATAAATCAGCACATTTTATTTTGTAGTTTTACAGTGTTGCTTGATAAACATTTTACCTAACAAAAAAAGA**

***Sse_intron3’* AATTAGCAACATAAATC---------------------TTACAGTGTTGCTTGATAAACATGTAACCTAACAATAAAATG**

2250 2260 2270 2280 2290 2300 2310 2320

....|....|....|....|....|....|....|....|....|....|....|....|....|....|....|....|

***Sse_intron3* ACACATTTTTTGTGAATTAATTAATAAACAAATATGTTTACCTATTTAAACAGTAACACAATATTAATTTATAGAAAAGA**

***Sse_intron3’* AACAATTTCTT-----CTTGTGAATTAATTAATATGTTTCCCTATTTACACAGTGACACAATATTGATTTATAGAAAGGA**

....|...

***Sse_intron3* CTACTACT**

***Sse_intron3’* --------**


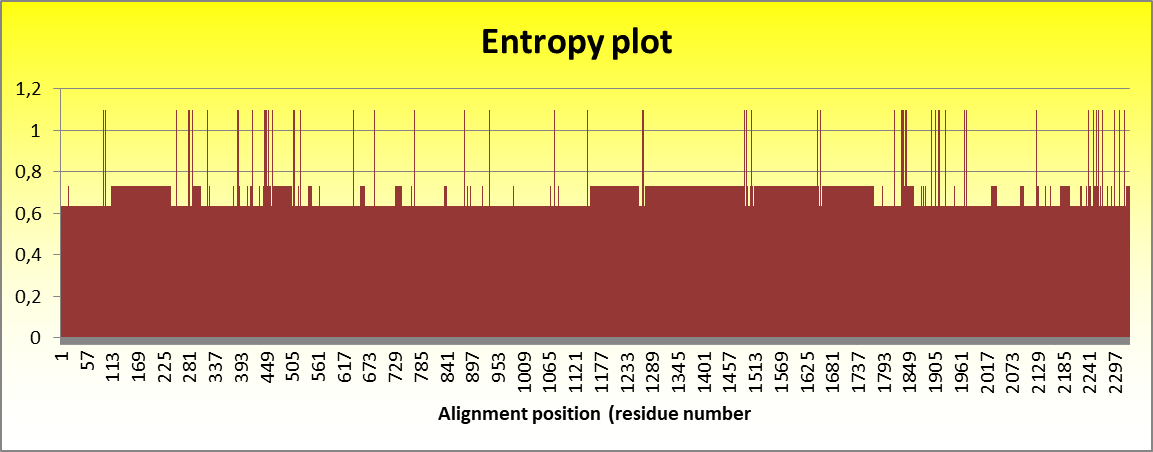

Supplement: S6 Fig — (A) Introns 1 and 1’ alignment and their plot of entropy; (B) Introns 2 and 2’ alignment and their plot of entropy; (C) Intron 3 and its duplicated region in intron 3’ alignment and their plot of entropy. (DOCX) [file pone.0241518.s010.docx]
