# Supplementary material for: The genomic structure of the highly-conserved dmrt1 gene in Solea senegalensis (Kaup, 1868) shows an unexpected intragenic duplication
Source: PLoS One. 2020 Nov 2;15(11):e0241518. doi: 10.1371/journal.pone.0241518 (PMC7605655; doi:10.1371/journal.pone.0241518)

**S7 Fig:** **Analysis or transposable elements in *S. senegalensis* *dmrt1* gene**. Upper figure shows the sequence of a cluster obtained after Graph-based clustering and characterization of repetitive sequences from 454 Roche NGS data of *Solea senegalensius* BAC clon 48K7. SINE sequence (SINE2-1B_DR) obtained after cluster BLAST search in Dfam database, is displayed in bold and underlined. Lower figure displays graph layout derived from a read cluster indicative of *S. senegalensis* repetitive sequence is displayed: Single reads are represented by nodes and their sequence overlaps by edges. This SINE element is located flanking the duplicated region in *dmrt1* gene.

>CL71Contig1

CAATAATAAAAACAAATAATAATGTGGAATGTGCATTAAATTAAAACATCAAAAAAAAAA

AAAAAGGCAGGTGTATATTATAAAGTCTATAACAATAACGTTAGACAGCTGGGCCTTGGA

TCTCCTCTGTATATTCTGTATTATCATTTATATTCTCTGAAAAATGGCCAAATTCTGGCA

GGGTATGTTAACTTATGAGCACAAATGTAGCTATTTACATCATTAAGTTTGTTTTACTTC

GATGCATCTTTATAAATTTGCCTTTTCACAGGTTGTTTTGAAGAAAATGGAAAATCCGTG

AAGAAGAAATATGTAACAGCTCTCAACGCATTCAATTGTAGAGTTTTAGGACAGGTAAAG

GAGAAAGAGTGGGAACACTTTTTCAGGTGACAACAAACTGCTTATTTTTTATTCTCAATA

AATATCAGTTTTGTTTATTTGTGAGCTCTTCACGCGCATACAAACAAAATGCCCCCTTGA

ATTTGAATAACCATAACGCGTCGCCTTCCCAGCCTGACAGCTTCACTGAAAATACCATAA

ATCACTAAGGAGGATGGCTAAATTATGCTAAAGAGTGCTAACTGATTAGCATTAATGTGC

TCACTTTCTCCTTCAAGCACTGAGGCATAACAGTTTGTTCTTCTGCCGACAGACAATACC

ATTAACCTTTGCAATTCAGACTCTGCTGTGCACTGGCAGGTTAGCGGAGGCAGTGACCCC

AAAACTAGATGTTGCTATTGAGAGGTGGGCGAGCGGGGTGAGTGTGGGGTGGCCAAACGT

TCTTCATAAAAAAAACAATCCACCTCATCAGAGAACACTGTTGAGTTATAGATCTAATTT

GCTTCACGCACAAGCTCATCTCATTTGACACAACAGGGATACATTCAAGCATAAATCTGT

GTGAATATTCTGAGAGAAACGTGTGTGTGTGTGTACCCTGGTGTGTCCTAGGTTATAAGA

CATTAAAGCAGACACGACCATCAATCTTTGAAAAACATAAAGAAATAAAAGTTTATGTAA

TTGACTCAAAGATGCAAAGAAAACAAACCTCTCACATTCACAAGGTTGCCCACATGTGCA

AAGTGAGGCTCTCTCTTTCTGTTGCATGTAAAAAATGTTATATGAATTTTTAGAGTTTCC

TTCATTTTCATCACTTTGTTTTCATTTTGTTTTTACAGTTTTATTCTGTAAGTCCTTTCT

ATAAATCAATATTGTGTCACTGTGTAAATAGGGAAACATATTAATTAATTCACAAGAAGA

AATTGTTCATTTTATTGTTAGGTTACATGTTTATCAAGCAACACTGTAAGATTTATGTTG

CTAATTTTAATTTTTTCCACTGTT**TTTTTTACTTGTTTAAATGTGTTTAAGTCTACTTTT**

**AGGGCACTTTGGCCTTCATTCATAAGCTGACAGCACAGACAAATGACAGCGAAAAAAGGG**

**CGAGTGAAGGGCGTGATGTGCAACATAAGCCTGGAATAGAGCACGGCCTGGGCCGCTTTT**

TCTGCCCGAACCCGACATGAAC


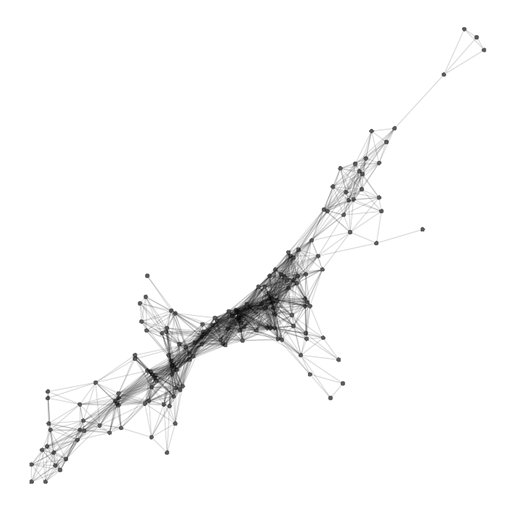

Supplement: S7 Fig — Upper figure shows the sequence of a cluster obtained after Graph-based clustering and characterization of repetitive sequences from 454 Roche NGS data of Solea senegalensius BAC clon 48K7. SINE sequence (SINE2-1B_DR) obtained after cluster BLAST search in Dfam database, is displayed in bold and underlined. Lower figure displays graph layout derived from a read cluster indicative of S. senegalensis repetitive sequence is displayed: Single reads are represented by nodes and their sequence overlaps by edges. This SINE element is located flanking the duplicated region in dmrt1 gene. (DOCX) [file pone.0241518.s011.docx]
